# Heterojunction Nanomedicine

**DOI:** 10.1002/advs.202105747

**Published:** 2022-02-17

**Authors:** Chao Pan, Zhuo Mao, Xue Yuan, Hanjie Zhang, Lin Mei, Xiaoyuan Ji

**Affiliations:** ^1^ Academy of Medical Engineering and Translational Medicine Medical College Tianjin University Tianjin 300072 China; ^2^ Tianjin Key Laboratory of Biomedical Materials Key Laboratory of Biomaterials and Nanotechnology for Cancer Immunotherapy Institute of Biomedical Engineering Chinese Academy of Medical Sciences and Peking Union Medical College Tianjin 300192 China

**Keywords:** catalytic therapy, electron‐hole pairs, heterojunction, nanomedicine, semiconductor

## Abstract

Exogenous stimulation catalytic therapy has received enormous attention as it holds great promise to address global medical issues. However, the therapeutic effect of catalytic therapy is seriously restricted by the fast charge recombination and the limited utilization of exogenous stimulation by catalysts. In the past few decades, many strategies have been developed to overcome the above serious drawbacks, among which heterojunctions are the most widely used and promising strategy. This review attempts to summarize the recent progress in the rational design and fabrication of heterojunction nanomedicine, such as semiconductor–semiconductor heterojunctions (including type I, type II, type III, P—N, and Z–scheme junctions) and semiconductor–metal heterojunctions (including Schottky, Ohmic, and localized surface plasmon resonance–mediated junctions). The catalytic mechanisms and properties of the above junction systems are also discussed in relation to biomedical applications, especially cancer treatment and sterilization. This review concludes with a summary of the challenges and some perspectives on future directions in this exciting and still evolving field of research.

## Graphical abstract

This review summarizes the recent progress in the rational design and fabrication of heterojunction nanomedicine. The catalytic mechanisms and properties of applied heterojunction systems are also discussed in relation to biomedical applications, especially cancer treatment and sterilization. This review concludes with a summary of the challenges and perspectives on future directions in this evolving field of research.

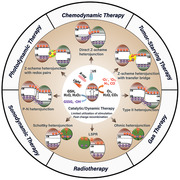

## 1 Introduction

Catalysis, as a utility strategy, is closely related to our survival and development because of its irreplaceable ability in controlling the rate of chemical reactions by decreasing the activation energy. In many aspects, catalysis has expressed its crucial importance in fields, for example, organic decomposition^[^ ^1^ ^]^ and energy regeneration,^[^ ^2^ ^]^ which provide the necessary conditions for our daily life. In the past years, catalysis technology has been wildly used to solve medical problems by virtue of its unique and excellent properties, providing new inspiration for the treatments of diverse pathological abnormalities. This new and promising field is called catalytic medicine in which non‐toxic catalysts instead of traditional drugs are employed for the treatment of diseases by catalyzing the reaction at the disease site to produce therapeutic products. According to the operation mode and treatment principle, catalytic therapy can be divided into two categories: endogenous catalytic therapy and exogenous stimulation catalytic therapy. Fenton or Fenton‐like reactions catalyzed by reductive metal ions or metal nanomaterials are representative of endogenous catalytic therapy, which is also called chemodynamic therapy (CDT).^[^ ^3^ ^]^ In Fenton or Fenton‐like reactions, hydroxyl radicals (·OH) derived from the decomposition of hydrogen peroxide are the main therapeutic product to eliminate tumor cells or pathogens in this treatment mode, which is independent of extra stimulation. However, the therapeutic efficacy is hindered by the monotonous catalytic substrate type with limited content at the lesion site.^[^ ^4^ ^]^ By virtue of the cooperation of various exogenous stimulants and catalysts, exogenous stimulation catalytic therapy, including photodynamic therapy (PDT),^[^ ^5^ ^]^ sonodynamic therapy (SDT),^[^ ^6^ ^]^ and other exogenous stimulation‐excited dynamic therapies,^[^ ^7^ ^]^ has the advantages of diverse catalytic substrates, high catalytic efficiency, and low limitation by the internal microenvironment. According to the mechanism of catalytic therapy, the physical and chemical properties of the catalyst are the key to the success of catalytic therapy.

**Figure 1:**
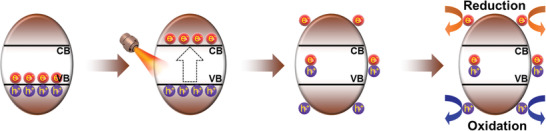
Functional progress of semiconductor photocatalysts.

Many emerging single semiconductor nanomaterials with improved biocompatibility, higher catalytic efficiency, and unique band structure have been prepared to enhance the generation of reactive oxygen species (ROS) from oxygen (O_2_) in a rational and simple way.^[^ ^8^ ^]^ The semiconductors are mostly crystals, and their valence electrons can be transferred between adjacent atoms and are affected by the potential field of the surrounding atoms due to the close arrangement of the constituent atoms, which results in splitting of energy levels and formation of energy bands. Analysis of the band structures of semiconductors is necessary, as they are the critical factors that determine a variety of the semiconductor properties. As shown in **Figure** **1**, a forbidden band without energy level is located between the energetically lower valence band (VB) and the energetically higher conduction band (CB). Due to the narrow forbidden band of semiconductors, the electrons in the VB can be transferred into the CB after absorption of exogenous energy (e.g., light, ultrasound, X‐rays). As a result, holes are left in the VB, which is a prerequisite for catalysis, and the separated carriers can take part in redox reactions.^[^ ^9^ ^]^ Based on this, semiconductor‐mediated catalytic therapy involves at least the following steps: a) Accumulation. It is essential for catalysts to be targeted and enriched at the tumor site to reach the therapeutic dose. b) Excitation. Stimulation provides enough energy for electrons to overcome the bandgap (*E*g) of the therapeutic agents and thus induce electron–hole pairs. c) Migration. After excitation, some excited carriers migrate to the surface of the material, while most of them recombine and release energy. d) Reaction. Electrons and holes at the surface undergo redox reactions with surrounding reactants to form ROS, such as superoxide anion (·O_2_^−^) (O_2_ + e^−^ → ·O_2_^−^, −0.33 eV vs normal hydrogen electrode (NHE)) and ·OH (OH^−^ + h^+^ → ·OH, 1.99 eV vs NHE), leading to irreversible permanent damage and apoptosis of tumor cells.

Semiconductors exhibiting a bandgap with more positive VB potential and more negative CB potential are promising candidates to expand the pool of substrates and products for cancer treatment.^[^ ^9a^ ^]^ On the other hand, such a band structure with a large bandgap brings about the difficulties of electron–hole pair separation and requirement on high energy light. Due to this contradiction, various and efficient ROS generation methods for single semiconductors can be hardly achieved at the same time. To overcome these disadvantages, various methods have been proposed, including surface modification,^[^ ^10^ ^]^ element doping,^[^ ^11^ ^]^ control of morphology,^[^ ^12^ ^]^ and construction of heterojunctions. Considering the efficiency and feasibility, the construction of heterojunctions using different photocatalysts is undoubtedly the most promising strategy to improve the photocatalytic ability. The band structures of catalysts can be tailored by reasonably designing the heterostructure. Compared with single semiconductors, heterojunction structures are more feasible and effective in applying external light with wavelengths in the biowindow for photoexcitation and inhibit the recombination of photoinduced electron–hole pairs.^[^ ^13^ ^]^ Apart from these advantages, heterojunctions containing various compounds have many unique functions while retaining the properties of a single component.^[^ ^14^ ^]^ Hence, in addition to the benefits in energy supply,^[^ ^15^ ^]^ pollutant disposal,^[^ ^16^ ^]^ and photodetectors optimization,^[^ ^17^ ^]^ fabricating appropriate heterojunctions also goes a long way towards the treatment of diseases, especially cancer treatments. As one of the biggest killers of human life and health, cancer urgently needs effective, safe, and reliable treatment strategies. Traditional treatment methods, such as surgery, chemotherapy, radiotherapy (RT), etc., inevitably bring great side effects to patients while treating cancer. Catalytic therapy, which replaces traditional drugs with safe and non‐toxic catalysts, achieves specific treatment of cancer by generating therapeutic products in situ in tumor tissues and greatly reduces the side effects of cancer treatment. However, due to the limited catalytic efficiency of single catalyst, the shielding effect of biological tissue on excitation energy source, and the complex inhibitory microenvironment of tumor tissue (hypoxia, high glutathione expression, etc.), the therapeutic efficiency of traditional catalytic therapy is seriously affected. Heterojunction nanomedicine can not only effectively inhibit excited electron‐hole recombination, improving the effective utilization efficiency of external excitation energy, but also contain multiple reactive active sites, expanding substrate selectivity and effectively regulating tumor inhibition microenvironment. Therefore, using heterojunction technology, development of novel and efficient heterojunction nanomedicine can achieve efficient and safe catalytic treatment of cancer.

**Scheme 1:**
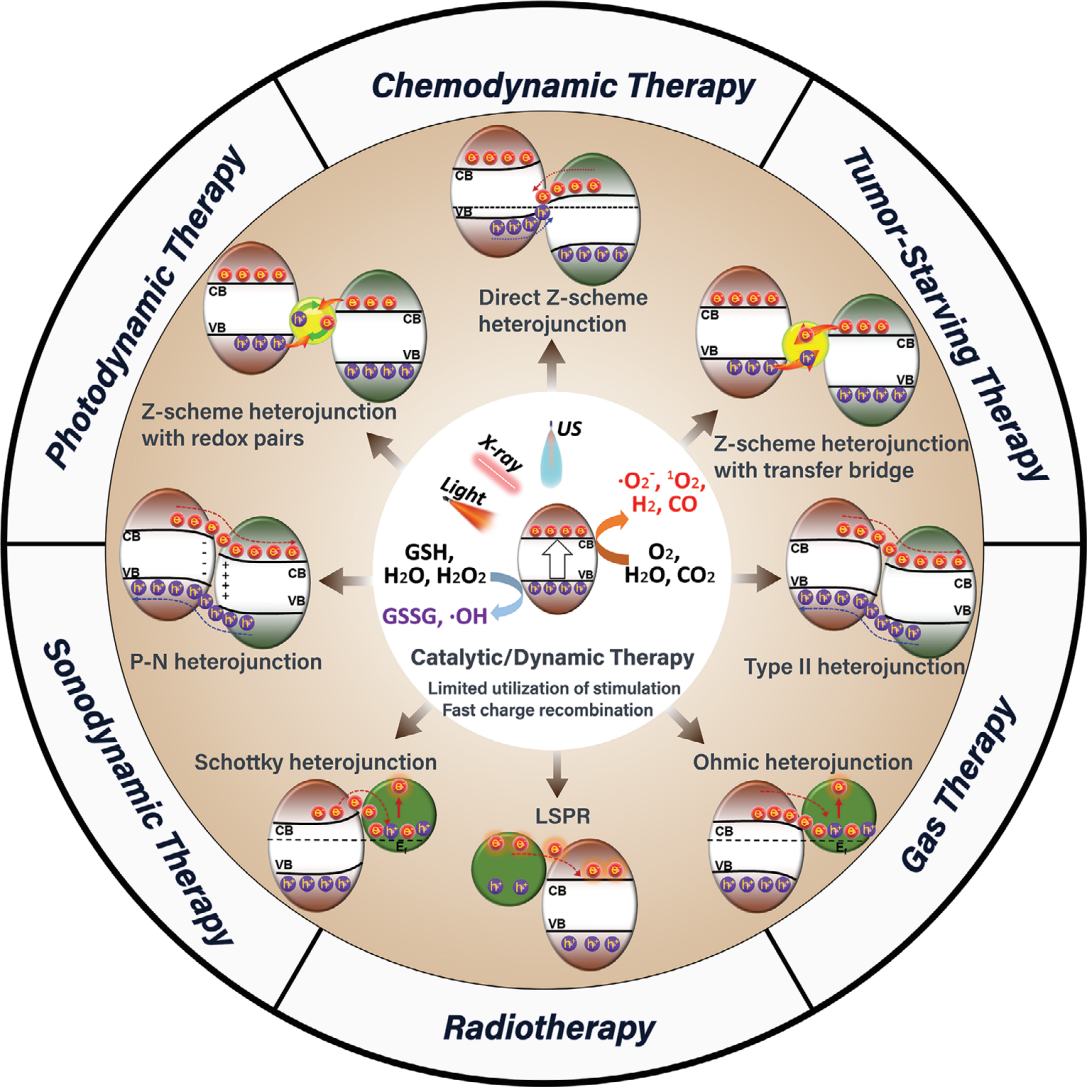
A schematic summarization of the classification, properties, and biomedical applications of heterojunction nanomedicine.

This review article focuses on the specific principles and preparation methods of heterojunctions (**Scheme** **1**). In addition, the recent design and application of various heterojunctions in biomedicine are introduced. Then, the optimization of existing heterojunctions as well as future applications and potential heterojunctions in other therapeutic approaches are presented, and prospects are discussed. The introduction of heterojunctions into biomedicine will greatly accelerate the development of multiple treatments, and we hope this review can provide some ideas to promote the further development of heterojunctions as high‐efficiency “medicine” in the field of biomedicine.

## 2 Working Principles of Different Heterojunction Photocatalysts

As promising candidates for overcoming the drawbacks of single‐component catalysts, heterojunction catalysts are formed by coupling two different functional materials. According to the conductivity of the materials, heterojunctions can be classified into two types: a) semiconductor–semiconductor (S–S) heterojunctions and b) semiconductor–metal (S–M) heterojunctions.

### 2.1 S–S Heterojunction

**Figure 2:**
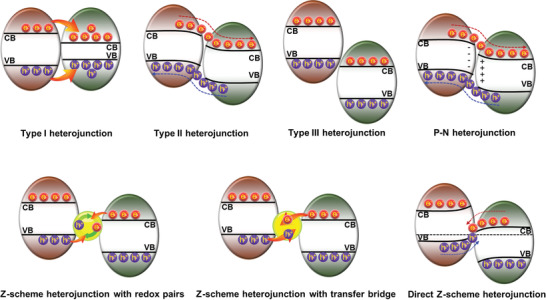
Schematic illustration of the photocatalytic principle of S–S heterojunctions.

The formation of semiconductor heterojunctions is a promising strategy for the preparation of high‐performance catalysts for the full conversion of light. Through surface engineering, such as doping and defect engineering, various intrinsic p‐ or n‐type semiconductors with different mechanical, physical, and chemical properties can be fabricated,^[^ ^18^ ^]^ providing abundant raw materials for the development of S–S heterojunctions. In general, the positions of the electronic band edges and the work functions of two semiconductors are important metrics to determine the electron transfer mechanism of a semiconductor heterojunction, which directly affects its catalytic performance.^[^ ^19^ ^]^ Based on the transfer mechanism of photoinduced electrons,^[^ ^20^ ^]^ S–S heterojunctions can be divided into type‐I, type‐II, and Z‐scheme heterojunctions (**Figure** **2**).

In type‐I heterojunctions, the energy band structures of the two catalyst systems are nested.^[^ ^21^ ^]^ As shown in Figure 2, the VB and the CB of the narrow‐bandgap photocatalyst are both located in the forbidden band of the wide bandgap photocatalyst. Since the band edges of narrow bandgap photocatalysts are lower upon light irradiation, the photoinduced electrons and holes will be transferred from the wide bandgap photocatalyst to the CB and VB of the narrow‐bandgap photocatalyst, respectively. This electron transfer route facilitates the recombination of the photoinduced carriers,^[^ ^22^ ^]^ which is beneficial for potential applications in light‐emitting devices^[^ ^23^ ^]^ but not conducive to photocatalytic reactions.

In type II heterojunction photocatalysts, the VB and CB edges of one semiconductor (catalyst I) are higher than those of the second semiconductor (catalyst II). The Fermi level of catalyst I is lower than that of catalyst II. Due to the rearrangement of the Fermi levels after contact of the catalysts, catalyst II acts as an electron donor to transfer electrons to catalyst I. Consequently, the energy bands of catalyst I bend downwards, while the energy bands of catalyst II bend upwards at the semiconductor interface. Under light irradiation, the photoinduced electrons and holes accumulate in the CB of catalyst II and the VB of catalyst I. Compared with type I heterojunctions, the photoinduced electrons and holes in type II heterojunctions are trapped in two spatially separated potential wells, which effectively increases the lifetime of the photoinduced carriers.^[^ ^24^ ^]^ However, the increase in the lifetime of photoinduced carriers is at the expense of a reduced redox capacity of the two semiconductor catalysts. In addition, the migration of photoinduced carriers is inhibited by the existing photoinduced carriers in another phase.

Type III heterojunctions are formed when the VBM of one photocatalyst is higher than the CBM of the second photocatalyst.^[^ ^25^ ^]^ As shown in Figure 2, the bandgaps of these two photocatalysts are completely separated. Therefore, photoinduced carrier separation and migration hardly occur between the two phases,^[^ ^26^ ^]^ and the efficiency of the photocatalytic reactions are thus not improved.

Inspired by natural photosynthesis, which realizes the electron transport by connecting two different photocatalytic systems via an electron transfer medium, A. J. Bard first proposed the liquid‐phase Z‐scheme heterojunction photocatalyst in 1979.^[^ ^27^ ^]^ The liquid‐phase Z‐scheme heterojunction photocatalyst consists of two semiconductors acting as catalytic centers and free redox mediators (Fe^3+^/Fe^2+^, IO^3−^/I^−^, etc.).^[^ ^28^ ^]^ As illustrated in Figure 2, photoinduced electron and hole pairs are generated in catalyst I and catalyst II under light irradiation. Then, the photoinduced holes in the VB of catalyst I and the photoinduced electrons in the CB of catalyst II are consumed by free redox ion pairs. In contrast, the photoinduced electrons in the CB of catalyst I with higher reduction potential and the photoinduced holes with higher oxidation potential in the VB of catalyst II will undergo redox reactions with the substrates. In addition, for the effective separation of photoinduced electron–hole pairs, the solid phase Z‐scheme heterojunction also retains the original strong redox capability of the semiconductors. However, shortcomings, such as a limited application environment, low electron transfer efficiency, light‐shielding effect, and serious side reactions, limit the further application of liquid‐phase Z‐scheme heterojunctions in the field of catalysis.^[^ ^28^ ^]^

To overcome these shortcomings of liquid‐phase Z‐scheme heterojunction photocatalysts, all‐solid‐state Z‐scheme heterojunction photocatalysts were subsequently developed. Conductive materials (e.g., precious metals, graphene) were adopted as electron transfer mediums in all‐solid‐state Z‐scheme heterojunction photocatalysts.^[^ ^29^ ^]^ Although the solid electron transfer medium expanded the application of Z‐scheme heterojunctions, the high cost of precious metals and difficulties in the design are remaining problems.

In 2001, Grätzel et al. first coupled nano‐sized transition metal oxides (WO_3_ or Fe_2_O_3_) with dye‐sensitized TiO_2_. They found that photoinduced holes in the VB of WO_3_ or Fe_2_O_3_ can effectively catalyze the oxidation of H_2_O, while the photoinduced electrons accumulate in the CB of TiO_2_.^[^ ^30^ ^]^ Since then, Z‐scheme heterojunctions without electron mediators have gradually entered the field of vision of researchers. Yu et al. reported a *g*‐C_3_N_4_/TiO_2_ Z‐scheme heterojunction in 2013.^[^ ^31^ ^]^ This type of Z‐scheme heterojunction was reported for the first time; it does not require an electron mediator and was named direct Z‐scheme heterojunction. Direct Z‐scheme heterojunctions, which are formed by two semiconductors that are in direct contact, do not require an additional electron transfer medium^[^ ^32^ ^]^ but still effectively follow the Z‐scheme electron transfer mechanism. The basic design principle of direct Z‐scheme heterojunctions is that the two semiconductors have a staggered gap, and the semiconductor with the higher potential (catalyst I) has a lower work function than the semiconductor with the lower potential (catalyst II).^[^ ^33^ ^]^ After contact with the two semiconductors, the electrons of catalyst I are transferred to catalyst II until the two Fermi levels reach equilibrium. Consequently, the bands of catalyst I bend upwards, while the bands of catalyst II bend downwards, providing the basis for the combination of the photoinduced holes in the VB of catalyst I and the photoinduced electrons in the CB of catalyst II through the tunneling effect. In addition, the internal electric field generated by the rearrangement of the Fermi level can further promote the combination of the above photoinduced electron–hole pairs. Moreover, the internal electric field effectively inhibits the combination of the photoinduced electrons on the CB of catalyst I with high reduction potential and the photoinduced holes in the VB of catalyst II with high oxidation potential. As a result, the direct Z‐scheme heterojunction possesses not only a wide catalytic substrate range but also a high catalytic efficiency.

In practice, intrinsic semiconductors are artificially and accurately endowed with various physical and chemical properties through doping engineering to meet various application requirements. In particular, by doping a specific proportion of heteroatoms or introducing defects, properties related to the catalytic ability of the semiconductor, such as the carrier type, Fermi level, and band structure, are modulated to obtain ideal raw materials for the preparation of excellent heterojunction photocatalysts. According to the type of the major carrier, the modified semiconductors can be divided into n‐ and p‐type semiconductors. The major carriers in n‐ and p‐type semiconductors are electrons and holes, respectively. According to the conductivity type of the semiconductor, heterojunctions can be divided into homotype heterojunctions (n‐n type and p‐p type) and heterogeneous p‐n junctions.^[^ ^34^ ^]^ Since the two semiconductors in a homotype heterojunction have the same conductivity type, carrier diffusion caused by Fermi level rearrangement is unidirectional. It is worth noting that when an n‐type semiconductor is connected with a p‐type semiconductor, electrons in the n‐type semiconductor near the p‐n interface will diffuse to the p‐type semiconductor, forming a positively charged area. Furthermore, the holes in the p‐type semiconductor near the p‐n interface will diffuse to the n‐type semiconductor, forming a negatively charged area. Charge diffusion will continue until the Fermi levels of the n‐ and p‐type semiconductors are in equilibrium. Due to the carrier concentration gradient, a stronger internal electric field will eventually be generated in p‐n junctions, effectively promoting the separation of photoinduced electrons and holes.

### 2.2 S–M Heterojunction

**Figure 3:**
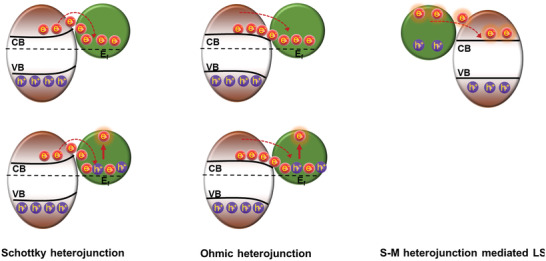
Schematic illustration of the photocatalytic principle of S–M heterojunctions.

The characteristics of metal–semiconductor contacts, which can be divided into Schottky junction and Ohmic contact, are related to the type of the semiconductor (i.e., n‐ or p‐type) and the relative work function between the metal and the semiconductor.^[^ ^35^ ^]^ In 1874, Schottky found that a surface potential‐energy barrier (Schottky barrier) exists in the semiconductor phase of some specific metal–semiconductor contacts (Schottky contacts; **Figure** **3**).^[^ ^36^ ^]^

In general, Schottky junctions are built by connecting a metal with higher work function (*Φ*) and an n‐type semiconductor with lower *Φ* or by connecting a metal with lower *Φ* and a p‐type semiconductor with higher *Φ*. Taking a Schottky junction formed by an n‐type semiconductor and a metal as an example, after the semiconductor and the metal come in contact, the electrons flow from the semiconductor with higher *Φ* to the metal with lower *Φ* until their Fermi levels reach equilibrium. Due to the limitation of the free electron density, a positively charged region forms in the semiconductor. Furthermore, the energy band of the semiconductor bends upward. Thereby, the rectification property of Schottky junctions makes it possible to control the production and flow of excited electrons, and the Schottky junction can serve as an electron trap to effectively promote the separation of excited electrons and holes. However, as a minority carrier in the n‐type semiconductor, the holes exhibit a decreased potential after the formation of a Schottky contact, resulting in a restricted oxidation ability.

Contrary to Schottky contacts, Ohmic junctions are formed by connecting an n‐type semiconductor with lower *Φ* and a metal with higher *Φ* or by connecting a p‐type semiconductor with higher *Φ* and a metal with lower *Φ*. Taking an n‐type semiconductor/metal Ohmic junction as an example, the band edges of the semiconductor bend downward after connection with the metal, which cannot effectively prevent the flow of excited electrons back to the VB of the semiconductor and the subsequent recombination of electron–hole pairs. Therefore, the Ohmic junction is not an ideal photocatalyst candidate due to its inefficient regulation of the photoinduced electron–hole separation.

If the frequency of the incident photon matches the vibration frequency of the overall free electrons of the precious metal nanoparticles in the Schottky contact, the metal will show a strong absorption of photon energy, which is the localized surface plasmon resonance (LSPR).^[^ ^37^ ^]^ The LSPR effect promotes the generation of photoinduced electrons with high energy, which are entitled hot electrons, in the noble metal. Then, the hot electrons cross the Schottky barrier and are injected into the semiconductor, while the photoinduced holes remain in the metal. At present, Au and Ag are the common plasmon‐resonance metals to alter the light absorption ability with resonance absorption peaks located at 580 and 530 nm, respectively.

Di et al. designed an Ag quantum dots‐modified BiOBr ultrathin nanosheet (Ag QDs/BiOBr) with high photocatalytic activity.^[^ ^37b^ ^]^ Using photoluminescence spectroscopy, they proposed another explanation of the mechanism of the transfer and separation of photoinduced electron–hole pairs based on the LSRP effect. In the Ag QDs/BiOBr Schottky junction, the holes generated in the Ag QDs via the LSPR effect can combine with excited electrons from the VB of BiOBr, leaving the excited hot electrons to catalyze the reduction of specific reaction substrates. At the same time, the holes left in the VB of the BiOBr nanosheet can catalyze the oxidation. Based on this mechanism, in the Schottky junction, the metal not only serves as an electron reservoir, but the electrons have also sufficient catalytic activity through the LSPR. In addition, the precious metal quantum dots with nano‐sized diameters dispersed on the surface of the semiconductor ultrathin nanosheet can provide more catalytic sites due to the larger specific surface area.

In addition to traditional metals, some materials that generate a large number of free carriers under certain circumstances can also be adopted as conductors for the development of S–M heterojunctions. For instance, the resistance of certain functional nanoceramics represented by ZrO_2_ and *β*‐Al_2_O_3_ will change with the changes in external fields such as mechanical force, temperature, and irradiation.^[^ ^38^ ^]^ Structural modification can further modulate the electrical conductivity of functional ceramics. Na‐*β*‐Al_2_O_3_ ceramics are a family of ceramics with fast ionic conductivity, which have been widely used as battery separators. Moreover, some layered nanomaterials, such as graphene and transition metal carbide as well as nitride or carbonitride (MXene), represented by Ti_2_C_3_, also have excellent superior conductivity,^[^ ^39^ ^]^ which undoubtedly provides a broad range of material candidates for the design of S–M heterojunctions.

## 3 Preparation Methods of Heterojunctions

At present, the devised and studied conventional heterojunction materials are mostly composed of two kinds of semiconductors. To improve the photoresponsiveness and enhance the separation efficiency of photoinduced carriers, it is vital to consider the semiconductor properties in detail, including morphology features, crystal structures, and band arrangement. Therefore, the appropriate construction method of heterojunctions plays a crucial role in optimizing their optical and catalytic performances. After decades of development, several strategies for the construction of heterojunctions have been established. In the following, typical methods for the construction of heterojunctions will be elaborated, including mechanical assembly, chemical deposition, surface oxidation, and the electron transfer chain‐mediated strategy.

### 3.1 Mechanical Method

As a common strategy, physical methods are applied to combine different semiconductors by mechanical forces, such as grinding, stacking, and stirring.

**Figure 4:**
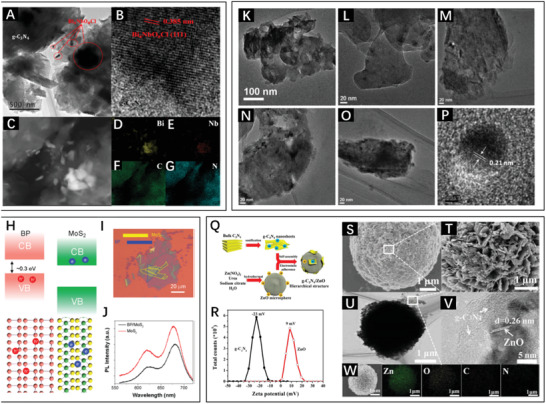
Characterization of g‑C_3_N_4_/Bi_4_NbO_8_Cl (6:1) by A) TEM, B) high resolution transmission electron microscopy (HRTEM), and C–G) EDX mapping.Reproduced with permission.^[^ ^40^ ^]^ Copyright 2018, American Chemical Society. H) Illustration of the BP/MoS_2_ heterojunction with type‐II band structure. I) Optical microscope image of the BP/MoS_2_ heterostructure (blue and yellow indicate the BP and MoS_2_ films, respectively). J) Photoluminescence spectra of MoS_2_ and BP/MoS_2_ exposed to irradiation at 514 nm. Reproduced with permission.^[^ ^41^ ^]^ Copyright 2019, Royal Society of Chemistry. TEM images of K) pure C_3_N_4_, L) 3%BP/C_3_N_4_, M) 5%BP/C_3_N_4_, N) 7%BP/C_3_N_4_, and O) 10%BP/C_3_N_4_. P) HRTEM images of 7%BP/C_3_N_4_. Reproduced with permission.^[^ ^42^ ^]^ Copyright 2018, Wieley‐VCH. Q) Schematic diagram of the synthesis of *g*‐C_3_N_4_/ZnO. R) Analysis of the zeta potentials of *g*‐C_3_N_4_ and ZnO. Characterization of 5%*g*‐C_3_N_4_/ZnO by S,T) FESEM, U) TEM, V) HRTEM, and W) EDS mapping. Reproduced with permission.^[^ ^43^ ^]^ Copyright 2018, Elsevier.

In the process of grinding, the materials’ grain sizes are decreased and the materials are mixed adequately to increase the interfacial interaction and strengthen the intermolecular force. You et al. reported g‑C_3_N_4_/Bi_4_NbO_8_Cl^[^ ^40^ ^]^ with a tight interface, which was prepared through a simple ball‐grinding method at high energy. The transmission electronic microscopy (TEM) images (**Figure** **4**A,B) showed dark Bi_4_NbO_8_Cl particles and the corresponding lattice fringe on g‑C_3_N_4_, illustrating the successful composition, which was confirmed by energy dispersive X‐ray (EDX) elemental mapping (Figure 4C–G).

The structures of most layered heterojunctions are held together by van der Waals forces resulting from the vertical stacking of semiconductors, which increases the contact area between different components for broad applicability. Nie et al. integrated exfoliated black phosphorus (BP) and MoS_2_ into a BP/MoS_2_ heterojunction with type‐II band alignment (Figure 4H) through direct stacking.^[^ ^41^ ^]^ In Figure 4I, the superimposed outlines demonstrate the integration of BP and MoS_2_ and that MoS_2_ coverage prevented the BP from fast degradation. To appraise the band structure and the heterostructure construction, the photoluminescence of MoS_2_ and BP/MoS_2_ was measured, and the spectra shown in Figure 4J reveal ≈30% reduction of the photoluminescence intensity attributed to formation of the BP/MoS_2_ heterojunction, suggesting the occurrence of type‐II charge transfer.

In addition, stirring is an effective way to enhance the interactions between materials to induce the mechanical assembly into heterojunctions. Kong et al. synthesized a BP/*g*‐C_3_N_4_ heterojunction through the modification of layered *g*‐C_3_N_4_ with BP quantum dots,^[^ ^42^ ^]^ and the corresponding TEM images are displayed in Figure 4K,L. In simple terms, the layered *g*‐C_3_N_4_ owned abundant free electrons and a large specific surface area to support the strong adsorption ability, which is beneficial to BP modification via stirring under vacuum.

Apart from adsorption, electrostatic attraction enables materials to assemble and form a contact surface. Nie et al. fabricated direct Z‐scheme *g*‐C_3_N_4_/ZnO microspheres via the preparation method shown in Figure 4Q to achieve an improved photocatalytic ability.^[^ ^43^ ^]^ In brief, the mixed dispersion of *g*‐C_3_N_4_ and ZnO was stirred to construct the heterojunction by electrostatic self‐assembly of positive ZnO and negative *g*‐C_3_N_4_ (Figure 4R). In contrast, the components are bound by electrostatic attraction more tightly, resulting in a more stable electronic structure. The characterization of this heterostructure is presented in Figure 4S–W.

This implementation is determined by the mechanical method, which is largely mediated by intermolecular forces and is a simple and effective method. However, it is difficult to form tight contacts in some heterojunctions only by physical forces, which could lead to shedding, cracking, or deformation. Thus, it is vital to explore other methods to stabilize heterojunction composites for photocatalysis in different systems.

### 3.2 Chemical Deposition

In the typical fabrication process of film heterojunction composites, the layered component is deposited onto the basement through chemical deposition, including liquid phase deposition (LPD), chemical vapor deposition (CVD), and electrochemical deposition (ECD). Compared with other strategies, the film prepared by chemical deposition is uniform and compact. Moreover, this bottom‐up approach provides outstanding repeatability and stability.

**Figure 5:**
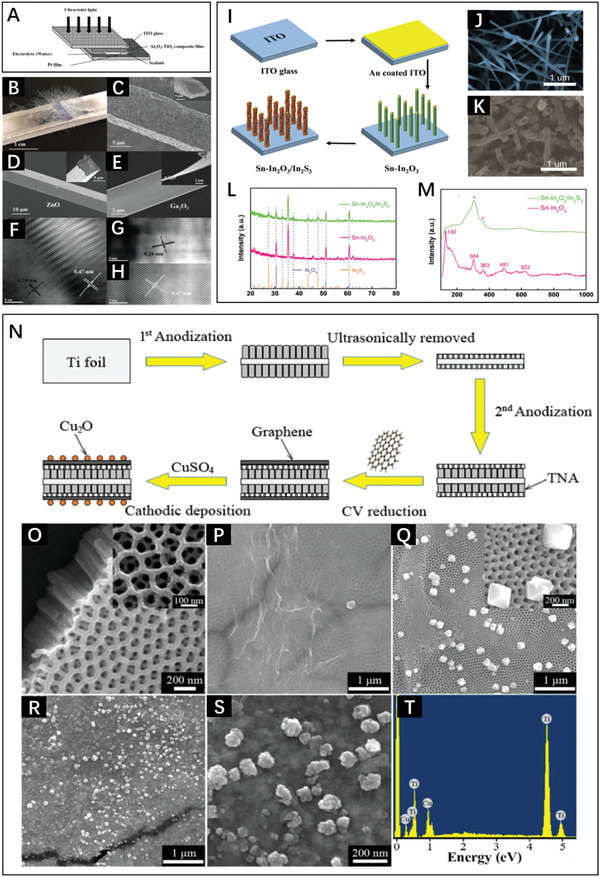
A) Illustration of the ultraviolet solid–liquid heterojunction photodetector. Reproduced with permission.^[^ ^46^ ^]^ Copyright 2020, Elsevier. B) Photograph of ZnO—Ga_2_O_3_ microwires with core–shell structure. C–E) Representative SEM images of ZnO—Ga_2_O_3_, ZnO, and Ga_2_O_3_ and the corresponding cross‐sectional images. HRTEM images of ZnO—Ga_2_O_3_ of F) the core and shell layers, G) core part, and H) shell part. Reproduced with permission.^[^ ^48^ ^]^ Copyright 2017, Wiley‐VCH. I) Scheme of the fabrication of the Sn—In_2_O_3_/In_2_S_3_ heterojunction. SEM images of J) Sn—In_2_O_3_ and K) Sn—In_2_O_3_/In_2_S_3_. Characterization of different samples by L) XRD and M) Raman spectroscopy. Reproduced with permission.^[^ ^49^ ^]^ Copyright 2021, Royal Society of Chemistry. N) Preparation method of Cu_2_O/graphene/ TiO_2_. Scanning electron microscope (SEM) images of O) TiO_2_, P) graphene/ TiO_2_, Q) Cu_2_O/ TiO_2_, and Cu_2_O/graphene/TiO_2_ at R) low and S) high magnification. T) EDS analysis of Cu_2_O/graphene/TiO_2._ Reproduced with permission.^[^ ^51^ ^]^ Copyright 2016, Elsevier.

LPD has been specially developed as a simple and efficient method for the formation of oxides films. Yuan et al. reported a mild route for the preparation of TiO_2_/boron‐doped‐diamond heterojunctions by LPD.^[^ ^44^ ^]^ These composites showed an average length of 2 µm, outside diameter of 100–150 nm, and wall thickness of 35–40 nm, exhibiting an improved photocatalytic ability for azo dyes. Zhang et al. reported WO_3_/TiO_2_ heterojunction films obtained by LPD,^[^ ^45^ ^]^ which expanded the response range to light to improve the photoelectrocatalytic activity for organic contaminants. In addition, in the field of photoelectric detection, Huang et al. employed the LPD method to deposit Al_2_O_3_ on TiO_2_ to optimize the photodetector performance (**Figure** **5**A),^[^ ^46^ ^]^ achieving an improvement of the optical responsivity of 56%.

In contrast to the LPD, in CVD, substances are introduced in the gaseous or vapor state for deposition on the substrate, providing products with high crystallinity and purity. Yu et al. employed CVD to fabricate uniform TiO_2_‐boron‐doped diamond heterojunctions^[^ ^47^ ^]^ with an improved ability in the photo‐mediated decomposition of dyes. In addition, through one‐step CVD, Zhao et al. designed highly crystallized ZnO—Ga_2_O_3_ microwires with core–shell heterostructures (Figure 5B–E),^[^ ^48^ ^]^ displaying potential application in space detection as solar‐blind photodetector. As shown in Figure 5F,G, two lattice spacings were detected, and the lattice interface between constituents was homogeneous and atomically sharp with few structural defects, which is beneficial to the photoresponsivity and response speed. In 2021, Ma et al. used CVD and a sulfidation method to prepare direct Z‐scheme Sn—In_2_O_3_/In_2_S_3_ heterojunction nanowires with 1D structure (Figure 5I).^[^ ^49^ ^]^ These nanowires not only avoided the lattice mismatch and the inherent interfacial defects between the two components but also provided effective channels to shorten the charge transmission path and diminish the recombination of carriers. As displayed in Figure 5J,K, Sn—In_2_O_3_ synthesized via CVD is smooth without obvious defects or grain boundaries. The X‐Ray diffraction (XRD) patterns (Figure 5L) show that the sharp characteristic diffraction peaks of Sn—In_2_O_3_ matched those of In_2_O_3_ well, demonstrating high crystallinity and phase purity, which was confirmed by Raman spectroscopy (Figure 5M).

Apart from the redox reaction employed in LPD and CVD, the external electric field can be utilized to prepare heterojunction composites in electrolyte solution via ion oxidization/reduction during ECD. This method has many advantages, such as diverse substrate shapes, controllable film thickness, and relatively low cost. Zhang et al. fabricated a Cu_2_O/ZnO/ITO heterojunction through two‐step ECD to optimize the electric properties of semiconductors.^[^ ^50^ ^]^ Moreover, to mitigate the effect of continuous CO_2_ emissions, Li et al. proposed a ternary Cu_2_O/graphene/TiO_2_ nanotube array heterostructure for CO_2_ reduction to methanol after ECD (Figure 5N).^[^ ^51^ ^]^ As presented in Figure 5O–S, the TiO_2_ nanotube array heterostructure formed via a two‐step anodization approach is highly ordered with a tube diameter of 30 nm and a tube length of ≈500 nm. After ECD, the graphene sheets and Cu_2_O nanoparticles (≈100 nm) were successfully deposited, which was confirmed by EDX (Figure 5T). As an effective method to fabricate films, ECD is often employed in conjunction with other strategies for heterojunction preparation. Wang et al. combined the strategies of ECD,^[^ ^52^ ^]^ heating treatment, and photoreduction to synthesize Bi/BiVO_4_, which manifested an improved photocurrent response and photoelectrochemical performance compared with pure BiVO_4_.

### 3.3 Surface Oxidation

Due to inspiration from photocatalytic water splitting via TiO_2_, a large number of oxide semiconductors with appropriate bandgap, high photocatalytic efficiency, and low cost have been reported. Perovskite oxides, such as PbZrO_3_, BaTiO_3_, PbTiO_3_, and SrTiO_3_,^[^ ^53^ ^]^ play an important role in photocatalytic water splitting and photovoltaic applications. Moreover, the narrow bandgap of the BiVO_4_ photocatalyst makes it a suitable candidate for photocatalytic applications in the visible light region,^[^ ^54^ ^]^ and various photocatalysts based on it have been explored. The TiO_2_‐based photocatalytic system has the advantages of easy operation, desired catalytic activity, and excellent stability.^[^ ^55^ ^]^ In addition, hematite (Fe_2_O_3_) is also valued for its advantages of high photocurrent density and strong photocorrosion resistance.^[^ ^56^ ^]^ In general, the optical performance of oxide‐based photocatalysts is outstanding, but the low mobility and high recombination of carriers hamper their further application to some extent. The introduction of heterojunctions allows the remodeling of band structures to broaden the responsive spectrum of the materials, and the electron migration paths are also optimized to improve the photocatalysis ability.

**Figure 6:**
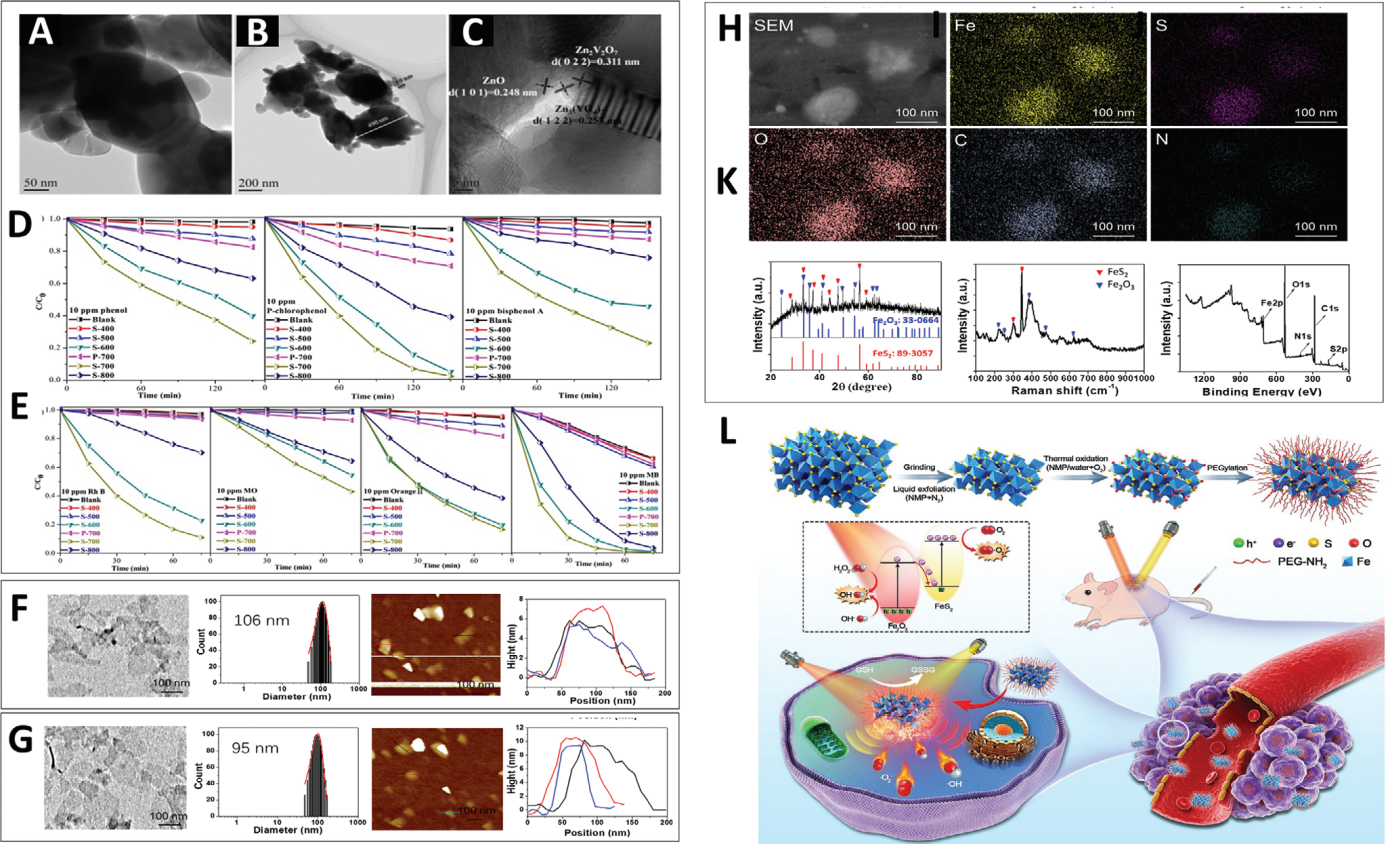
A–C) HRTEM images of Zn_3_(VO_4_)_2_/Zn_2_V_2_O_7_/ZnO nanosheets after treatment at 700 °C. Photocatalytic ability of as‐prepared samples in the degradation of D) phenols and E) dyes. Reproduced with permission.^[^ ^57^ ^]^ Copyright 2018, Elsevier. TEM images, size distribution, atomic force microscope (AFM) images, and thickness of F) TOPY NSs and G) TOPY‐PEG NSs. H) XRD spectra, I) Raman spectra J) X‐ray photoelectron spectroscopy (XPS) spectra, and K) EDS mapping of TOPY‐PEG NSs. L) Scheme of the preparation and cancer therapy of TOPY‐PEG NSs. Reproduced with permission.^[^ ^58^ ^]^ Copyright 2019, Wiley‐VCH.

Thus, surface oxidation has become one of the efficient strategies to modify and optimize the catalytic performance of photocatalysts. Zeng et al. designed the Z‐scheme ternary heterojunction system Zn_3_(VO_4_)_2_/Zn_2_V_2_O_7_/ZnO through the combination of the hydrothermal method and a calcination process.^[^ ^57^ ^]^ **Figure** **6**A,B shows that lots of small nanosheets were formed on the surface when the nanocomposite underwent partial phase transformation of Zn_3_(VO_4_)_2_ at 700 °C for 2 h. Analysis of the crystal planes based on the lattice fringes proved the existence of Zn_3_(VO_4_)_2_, Zn_2_V_2_O_7_, and ZnO (Figure 6C), which constitute the ternary heterojunction. During thermal oxidation, temperature control is a critical factor determining the photocatalytic effect. In a comparative study, Zn_3_(VO_4_)_2_/Zn_2_V_2_O_7_/ZnO nanosheets (after treatment at 700 °C (S‐700)) presented an improved photocatalytic degradation ability, which is displayed in Figure 6D,E.

In addition to calcination, ultrasonication is another efficient method for the introduction of surface oxides. In 2019, our group synthesized thermally oxidized pyrite nanosheets (TOPY NSs) with Z‐scheme heterojunction as an efficient photocatalyst for PDT.^[^ ^58^ ^]^ In short, we employed ultrasonication under different atmospheres to simultaneously achieve liquid‐phase exfoliation and thermal oxidation. This was not only beneficial for the formation of nanosheets but also overcame the shortcomings of oxide impurity formation in regular liquid‐phase exfoliation for the introduction of heterojunctions in photocatalysis. For further verification, a series of characterization methods was conducted. As shown in Figure 6F, the average size of TOPY NSs was 106 nm, and the thickness was 7 nm, providing numerous active sites for reactions. After coating with PEG–NH_2_ to impart biocompatibility and hydrophilicity, the size and thickness of the TOPY‐PEG NSs were altered to 95 and 10 nm, respectively (Figure 6G). XRD, Raman spectroscopy, and XRD presented in Figure 6H–J confirmed the successful construction of the TOPY‐PEG NS heterojunction with intimate contact between the Fe_2_O_3_ shell and the FeS_2_ core, which could accelerate the photogenerated electron transfer to facilitate ROS generation. Finally, energy dispersive spectrometry (EDS) mapping revealed a homogeneous distribution of Fe, S, O, C, and N in the TOPY‐PEG NSs, confirming that the heterojunction had a high oxide content and PEG attached to the surface of the TOPY‐PEG NSs (Figure 6K).

### 3.4 Electron Mediator

**Figure 7:**
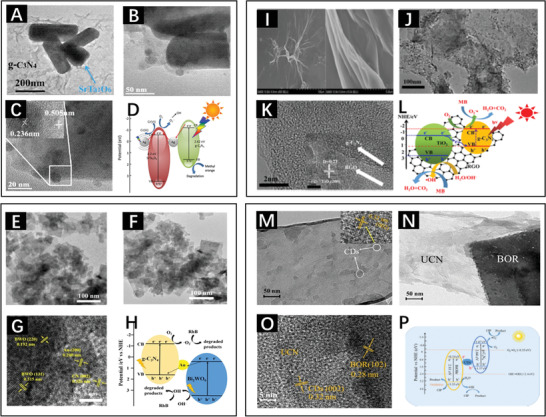
TEM images of A) SrTa_2_O_6_/*g*‐C_3_N_4_ and B) Ag‐SrTa_2_O_6_/*g*‐C_3_N_4_. C) HRTEM image of Ag‐SrTa_2_O_6_/*g*‐C_3_N_4_. D) Schematic diagram of the Ag‐SrTa_2_O_6_/*g*‐C_3_N_4_ heterojunction exposed to visible light irradiation. Reproduced with permission.^[^ ^59^ ^]^ Copyright 2015, Elsevier TEM images of E) BWO and F) CN/Au/BWO. G) HRTEM images of CN/Au/BWO. H) Diagrammatic sketch of the CN/Au/BWO heterojunction photocatalyst. Reproduced with permission.^[^ ^60^ ^]^ Copyright 2020, Elsevier I) SEM images of GO. J,K) HRTEM images of *g*‐C_3_N_4_‐RGO—TiO_2_. L) Mechanism of the photocatalytic degradation of MB by *g*‐C_3_N_4_‐RGO‐TiO_2_. Reproduced with permission.^[^ ^61^ ^]^ Copyright 2017, Elsevier TEM images of M) CDs/*g*‐C_3_N_4_ and N) BiOBr/CDs/*g*‐C_3_N_4_. O) HRTEM image of BiOBr/CDs/*g*‐C_3_N_4._ P) Illustration of photocatalysis mediated by BiOBr/CDs/*g*‐C_3_N_4_. Reproduced with permission.^[^ ^62^ ^]^ Copyright 2019, Elsevier.

In spite of a huge improvement in photocatalysis due to heterojunction construction, the electron mediator needs to be introduced into the system as a bridge to transfer carriers for further promotion of the photocatalytic efficiency, especially in the Z‐scheme heterojunction. With the development of heterojunctions, electronic mediators are also being optimized and upgraded. In contrast to the shortcomings of the liquid phase heterojunction system in the potential reverse reaction of redox pairs, which might abate the separation efficiency of charges in the contact area, the all‐solid heterojunction significantly facilitates the capability of carriers to cross the interface depending on the solid electron mediator. Noble metals, such as Ag, Au, and Pt, are regarded as good candidates due to their excellent electrical conductivity. Su et al. fabricated the ternary heterostructure Ag—SrTa_2_O_6_/*g*‐C_3_N_4_ with improved light absorption and photocatalytic activity.^[^ ^59^ ^]^ **Figure** **7**A shows a close contact of SrTa_2_O_6_ and *g*‐C_3_N_4_, and Figure 7B,C confirms the modification with Ag through morphology characterization and crystal structure analysis. Ag not only separated the electron–hole pairs via LSPR but also acted as a mediator to transfer the photogenerated electrons from the CB of *g*‐C_3_N_4_ to the CB of SrTa_2_O_6_ (Figure 7D). Similarly, Li and co‐workers modified the C_3_N_4_/Bi_2_WO_6_ (CN/BWO) heterojunction with Au,^[^ ^60^ ^]^ and the corresponding TEM images are shown in Figure 7E–G. As a mediator, Au in this heterostructure system facilitated the recombination of electrons in the CB of BWO and the holes in the VB of CN, causing higher redox potentials for ROS generation (Figure 7H).

Despite the excellent electron transfer performance of precious metals, high cost impeded further research. Carbon materials with low cost, such as graphene, are characterized by an excellent photoelectric performance, which is ascribed to the outstanding *π*‐electron transfer characteristics and the large specific surface area. Wu et al. inserted oxide graphene (RGO) into C_3_N_4_‐TiO_2_ to form *g*‐C_3_N_4_‐RGO—TiO_2_ nanoheterojunctions,^[^ ^61^ ^]^ as presented in Figure 7I–K. Notably, these nanocomposites with 10% RGO mass ratio displayed an optimized photocatalytic activity compared with the direct Z‐scheme C_3_N_4_‐TiO_2_ heterojunction (Figure 7L). This was attributed to the increase and tightness of the contact area, as well as the improved surface adsorption and reaction kinetics. Apart from RGO, carbon dots served as the mediator to inhibit the recombination of photocarriers and expand the light absorption range. Zhang et al. fabricated a BiOBr/CDs/*g*‐C_3_N_4_ heterojunction by a facile polymerization method,^[^ ^62^ ^]^ and the TEM images of this heterojunction are presented in Figure 7M–O. CDs, as the charge transmission bridge connecting BiOBr and *g*‐C_3_N_4_, not only enhance light absorption in the infrared region but also induce the immigration of electrons into the CB of BiOBr and holes into the VB of *g*‐C_3_N_4_ for photocatalysis on higher potential (Figure 7P). Interestingly, transformation of the heterojunction type through alteration in the direction of carrier migration can be achieved by the rational introduction of electron mediators.

Interestingly, in addition, to promote the separation of charges, the transfer direction of electrons could be changed by decoration of the mediators. Palanivel et al.^[^ ^63^ ^]^ inserted CD mediators into NiFe_2_O_4_/*g*‐C_3_N_4_ to prepare a ternary heterojunction via the simple wet chemical impregnation method. For the binary heterojunction, the photogenerated carriers accumulated at a relatively low position through type‐II transfer, which was insufficient to catalyze ·O_2_^−^ generation. In contrast, the Z‐scheme transfer of the photogenerated electrons after the introduction of CD excites electrons to a higher potential to participate in the production of ·O_2_^−^, improving the catalytic efficacy remarkably.

## 4 Application of Different Heterojunction Photocatalysts in Biomedicine

### 4.1 Type II Heterojunctions

Type II heterojunction is one of the most best‐known and widely used heterojunctions. For type II heterojunction, the band structures (VB and CB) of the two catalysts are interlaced, for example, the VB and CB edges of one semiconductor (catalyst I) are higher than those of the second semiconductor (catalyst II). The Fermi level of catalyst I is lower than that of catalyst II. Due to the rearrangement of the Fermi levels after contact of the catalysts, catalyst II acts as an electron donor to transfer electrons to catalyst I. Consequently, the energy bands of catalyst I bend downwards, while the energy bands of catalyst II bend upwards at the semiconductor interface. Under exogenous energy irradiation, the excited electrons and holes transfer spontaneously and accumulate in the CB of catalyst II and the VB of catalyst I. Compared with other heterojunctions, type II heterojunction can avoid the waste of catalytic energy caused by the recombination of excited electrons and holes by gathering excited electrons and holes in the CB of different catalysts.

**Figure 8:**
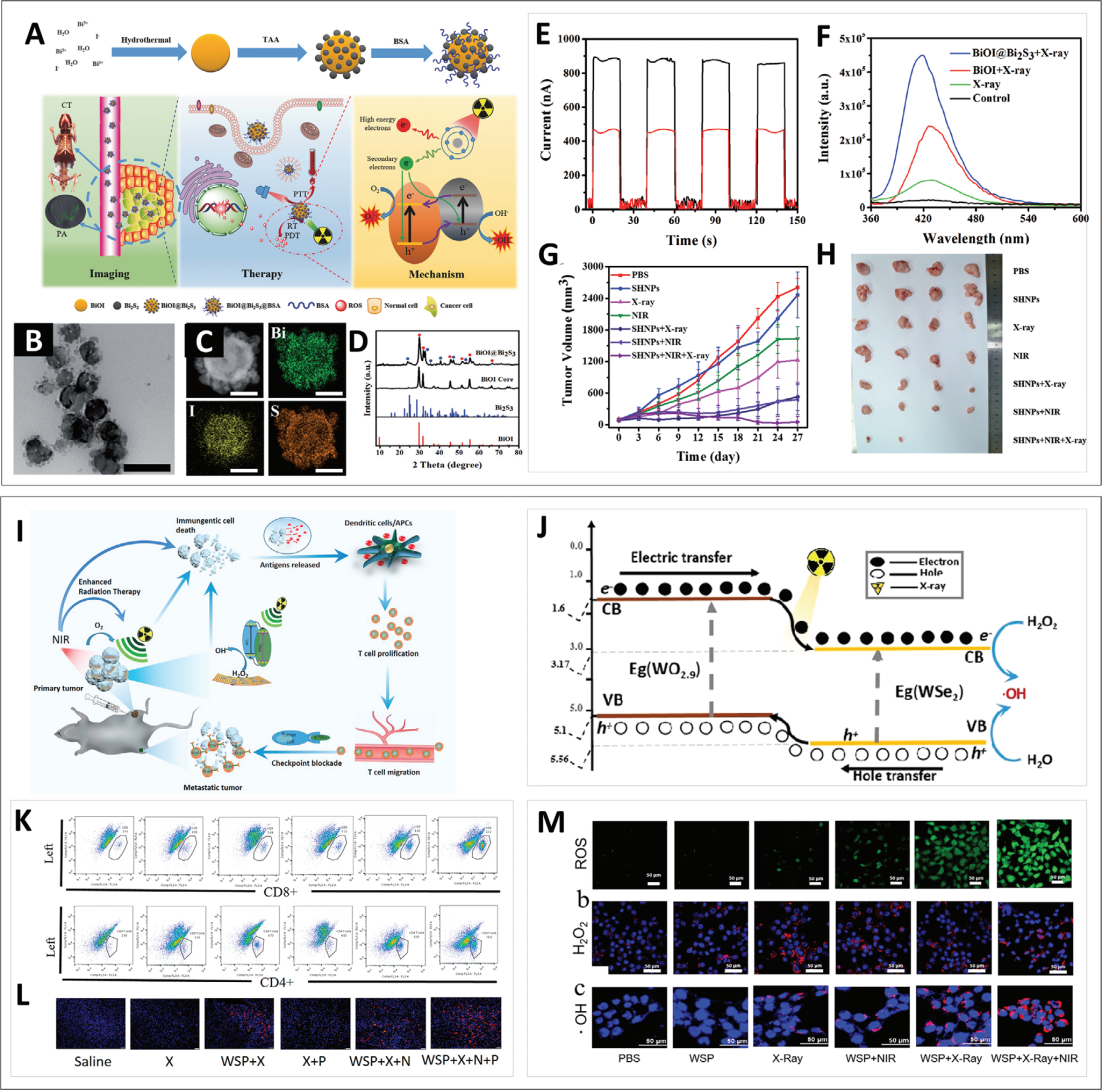
A) Illustration of BSA‐coated BiOI@Bi_2_S_3_ heterojunction‐mediated imaging, therapy, and the underlying mechanism. B–D) Characterization of BSA‐coated BiOI@Bi_2_S_3_ by TEM (scale bar: 200 nm), EDS (scale bar: 50 nm), and powder XRD. E) X‐ray‐triggered photocurrent responses of BiOI and BiOI@Bi_2_S_3_ with/without X‐ray irradiation. F) Fluorescence intensity of sodium terephthalate under different conditions. G) Tumor growth curves of mice during treatment. H) Representative photos of tumors in different treatment groups. Reproduced with permission. ^[^ ^64^ ^]^ Copyright 20217, Wiley‐VCH. I) Schematic diagram of WO_2.9_‐WSe_2_‐PEG‐mediated cancer therapy. J) Radiosensitization mechanism of WO_2.9_‐WSe_2_‐PEG. T cells in tumors in different treatments detected by K) flow cytometry and L) immunofluorescence staining. M) Confocal fluorescence images of ROS, H_2_O_2_, and ·OH in different treatment groups. Reproduced with permission.^[^ ^65^ ^]^ Copyright 2020, American Chemical Society.

Recently, Guo and co‐workers reported a strategy for the fabrication of BiOI‐based photocatalysts.^[^ ^64^ ^]^ In simple terms, after the preparation of BiOI via a hydrothermal reaction, nanoparticles with an average size of 100 nm were formed by anion exchange and BSA modification on the BiOI@Bi_2_S_3_ type II heterojunction (SHNPs; **Figure** **8**A,b). The composite structure of this heterojunction was verified through element analysis and XRD spectra (Figure 8C). In addition, this system with high‐Z elements could be excited by X‐rays for the purpose of RT and PDT. Specifically, due to the interlaced band structure and Fermi levels of BiOI and Bi_2_S_3_, under X‐ray irradiation, the excited electrons moved easily to the CB of BiOI, while the excited holes were transferred to the VB of Bi_2_S_3_ (Figure 8A). Then, the excited electrons and holes reacted with O_2_ and OH^−^, respectively, to generate ROS for cancer treatment. The photocurrent response of SHNPs under X‐ray irradiation shown in Figure 8E reveals that the SHNPs are X‐ray sensitive, which implies the higher yield efficiency due to the transfer of photogenerated carriers. As a result, X‐ray exposure of the BiOI@Bi_2_S_3_ type II heterojunction resulted in a superior ROS generation ability compared with that of pure BiOI, as indicated by the fluorescence change of the ROS probe sodium terephthalate (Figure 8F). In addition, the excellent photothermal (PT) conversion capability endowed SHNPs with the ability to ablate tumor cells. The results displayed in Figure 8G,H revealed the disappearance of the tumor in the SHNPs+NIR+X‐ray group, illustrating the desirable effect of synergistic treatments on the basis of SHNPs‐triggered RT/PDT/PTT. Moreover, after formation of the heterojunction, SHNPs were expected to act as computerized tomography (CT) and photoacoustic (PA) contrast agents to achieve a “one for all” cancer theranostic nanoplatform.

Similarly, Dong et al. reported a strategy for a combined therapy on the basis of WO_2.9_—WSe_2_ (Figure 8I).^[^ ^65^ ^]^ On the premise of the type II heterojunction construction, the X‐ray‐triggered charges moved in the direction shown in Figure 8J for the enhanced separation of electron–hole pairs, which led to the occurrence of cytotoxic ·OH facilitating immunogenic cell death (ICD). Furthermore, the PT effect of the WO_2.9_—WSe_2_ heterojunction was able to induce ICD, which improved the immunogenicity and triggered specific immune responses at the tumor site in synergy with checkpoint blockade immunotherapy (CBT). Through the analysis of tumor‐infiltrating T cells at remote tumor sites (Figure 8K), they found that the number of T cells increased during the treatment with WO_2.9_‐WSe_2_, and the strongest immune response was triggered after treatment with WO_2.9_‐WSe_2_ and anti‐PD–L1 antibody under NIR and X‐ray irradiation. This result demonstrated that heterojunction‐dependent RT/PTT/CBT plays a crucial role in both antitumor effect and immune response, which is in agreement with the immunostaining assay results (Figure 8L). In an in vitro assay (Figure 8M), WO_2.9_—WSe_2_ displayed the outstanding ability of ROS generation upon X‐ray and NIR irradiation. In vivo, the negligible tumor growth demonstrated the great inhibitory activity of WO_2.9_—WSe_2_ with irradiation and programmed death ligand‐1 (PD–L1) against primary and distant tumors. Taken together, these studies not only proved that a reasonably matched energy band would strengthen the therapeutic effect through the separation of charges but also confirmed the potential of the heterojunction in various treatments.

To further promote photocatalysis, a series of accessory compounds were added to the type II heterojunction system. Liu et al. introduced Au into CdSe/Bi_2_Se_3_ with type II band alignment to take advantage of the plasmon‐induced local field enhancement and hot electron injection for the enhancement of heat generation resulting from type II separation of charge pairs.^[^ ^66^ ^]^ In addition, Zhang and co‐workers integrated the upconversion particles NaYF_4_:Yb^3+^, Tm^3+^ into the Zn_2_GeO_4_:Mn^2+^ (ZGM)/*g*‐C_3_N_4_ heterojunction to match the irradiation frequency and the band structure of ZGM/*g*‐C_3_N_4_ for function enhancement of type II separation,^[^ ^67^ ^]^ which contributed to ROS production.

**Figure 9:**
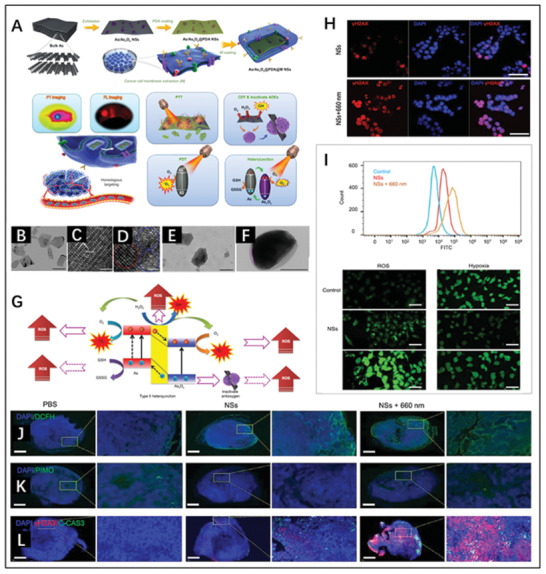
A) Schematic diagram of the fabrication of and tumor theranostics with As/As_x_O_y_@PDA@M NSs. B) TEM images of As/As_x_O_y_ NSs (scale bar: 100 nm). C) HRTEM images of As NSs (scale bar: 1 nm). D) HRTEM images of As/As_x_O_y_ NSs (red and blue circles indicate lattice fringes and As_x_O_y_ amorphism, respectively; scale bar: 1 nm). TEM images of E) As/As_x_O_y_@PDA NSs and F) As/As_x_O_y_@PDA@M NSs (scale bar = 100 nm). G) ROS burst induced by As/As_x_O_y_@PDA@M NSs. H) Confocal laser scanning microscopy (CLSM) images of MCF‐7 in different groups (scale bar: 50 µm). I) Generation of ROS and O_2_ in vitro. Detection of J) ROS and K) O_2_ generation in tumor tissue by DCFH and pimonidazole (PIMO) via fluorescence microscopy (scale bar: 1000 µm). L) Immunofluorescence staining of tumors in different treatments groups (scale bar: 1000 µm). Reproduced with permission.^[^ ^68^ ^]^ Copyright 2021, Springer Nature.

As an antioxidant widely present in tumor sites, glutathione (GSH) plays a significant role in the redox equilibrium of tumors. Notably, the strong reduction property of GSH hampers ROS‐induced cell death, leading to the inefficiency of most photocatalytic therapies. Given this issue, Ji et al. proposed type II‐based As/As_x_O_y_@PDA@M NSs for ROS burst,^[^ ^68^ ^]^ which were synthesized through ball grinding, liquid exfoliation, and further coating with polydopamine and cancer cell membrane (**Figure** **9**A). Figure 9B shows that the obtained As/As_x_O_y_ NSs had a layered structure with an average size of 93 nm. Owing to this, plenty of active sites were provided for the generation of photocatalytic singlet oxygen (^1^O_2_) and ·O_2_^−^. In addition, the presence of abundant oxygen vacancies on the surface induces the disproportionation of H_2_O_2_, which not only contributes to ·OH generation but also complements O_2_ to facilitate self‐enhanced PDT. On the other hand, ROS become more destructive because the photo‐triggered type II electron transfer resulted in the accumulation of sufficient holes for the oxidation of GSH, and the As_x_O_y_ NSs inactivated some main anti‐oxidases in the tumor site (Figure 9G). As shown in Figure 9H, irreversible DNA damage was caused in MCF‐7 cells treated with As/AsxOy@PDA@M NSs plus laser irradiation, which resulted from ROS burst and O_2_ generation (Figure 9I). A consistent conclusion was drawn based on further exploration in vivo (Figure 9J–L). In general, the synergism between the tripartite ROS generation and two main ROS consumption pathways improved the weak curative effect of the common type II heterojunction, providing a new cancer therapeutic strategy with superior efficacy and biosafety.

Although the catalytic activity increased due to the improved charge separation efficiency and the addition of adjuvants, this kind of type II charge transfer, similar with that of the type I heterojunction, decreases the redox potentials and redox capacity, resulting in a limited variety of therapeutic products. Moreover, it is physically unfavorable for the photoinduced electrons or holes to migrate through the type II heterojunction due to the electrostatic repulsion by the electron‐rich CB or the hole‐rich VB, respectively. Thus, it is necessary to further promote the development of heterojunctions.

### 4.2 Z‐Scheme Heterojunction

As mentioned before, based on the medium, Z‐scheme heterojunction could subdivide into three different subclasses, including Z‐scheme heterojunction with redox pairs, Z‐scheme heterojunction with transfer bridge, and Z‐scheme heterojunction without medium. Similar to type II heterojunction, the band structures (VB and CB) of the two catalysts are interlaced. However, the transfer path and mode of excited electrons and holes of Z‐scheme heterojunction were totally different from that of type II heterojunction. Specifically, the excited electrons at lower CB of catalyst II would be combined with the holes at lower VB of catalyst I, leaving excited electrons and holes at higher CB of catalyst I and higher VB of catalyst II.

**Figure 10:**
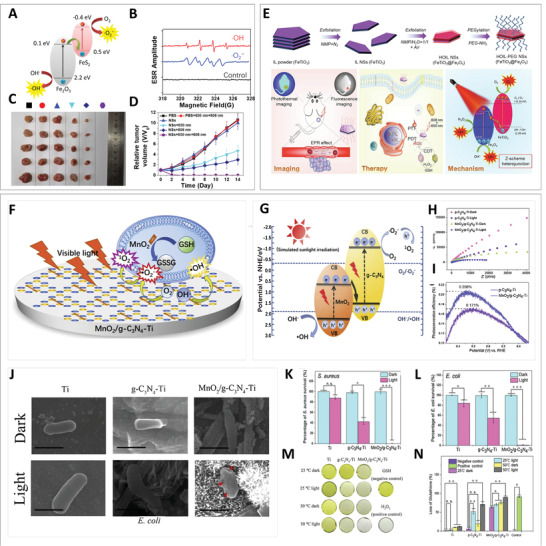
A) Electron transfer mechanism of TOPY‐PEG NSs. B) ESR spectra of ROS detection by TOPY‐PEG NSs. C,D) Therapeutic efficiencies of TOPY‐PEG NSs in different groups. Reproduced with permission.^[^ ^58^ ^]^ Copyright 2020, Wiley‐VCH. E) Illustration of the preparation and therapeutic principles of HOIL‐PEG. Reproduced with permission.^[^ ^69^ ^]^ Copyright 2020, Elsevier.F) Mechanisms underlying the antibacterial effect of MnO_2_/*g*‐C_3_N_4_‐Ti exposed to visible light. G) Electron transfer of MnO_2_/*g*‐C_3_N_4_‐Ti for ROS generation. H) EIS spectra and I) photoconversion efficiencies detected under different conditions. J) SEM images of *E. coli* in different groups (scale bars: 1 µm). Survival percentages of K) *S. aureus* and L) *E. coli* after co‐incubation with different samples under irradiation for 20 min. M,N) GSH consumption and the corresponding loss plot recorded under different conditions. Reproduced with permission.^[^ ^70^ ^]^ Copyright 2019, Elsevier.

Similar to natural photosynthesis, the Z‐scheme heterojunction can achieve effective photocatalytic reactions at high redox potentials in contrast to the type II heterojunction. Inspired by the above‐mentioned fundamental principles, Ji et al. synthesized TOPY NSs with Z‐scheme heterojunction via ultrasound‐assisted surface oxidation,^[^ ^58^ ^]^ as described above, as an efficient photocatalyst for PDT (**Figure** **10**A). To verify the function of the Z‐scheme heterojunction, ROS analysis was performed (Figure 10B). More specifically, 5,5‐dimethyl‐1‐pyrroline‐N‐oxide was used as a spin trapping agent for electron spin resonance (ESR) detection. Signals attributed to ·O_2_^−^ and ·OH indicated the formation of TOPY NSs with Z‐scheme heterojunction and the superior photocatalytic performance. In an in vivo assay (Figure 10C,D), TOPY NSs exhibited an enhanced therapeutic effect upon 650 and 808 nm laser irradiation compared with other groups owing to the optimized photocatalytic effect induced by the heterojunction. Notably, the synergism with the TOPY NS‐mediated Fenton reaction provided not only additional ·OH for CDT but also O_2_ supplement to alleviate hypoxia in the tumor site for PDT, which further improved the tumor‐killing activity.

To verify the universality of ultrasound‐assisted surface oxidation, highly oxidized ilmenite nanosheets (HOIL NSs) were prepared in a similar way.^[^ ^69^ ^]^ Such an excellent therapeutic effect was also presented by the system of HOIL‐PEG NSs. On the other hand, the potential of HOIL‐PEG NSs for fluorescence imaging and thermal imaging allowed the development of a multimodal imaging‐guided synergistic treatment (Figure 10E).

Recently, Wu et al. successfully prepared a Z‐scheme MnO_2_/*g*‐C_3_N_4_ heterojunction on Ti implants (MnO_2_/*g*‐C_3_N_4_‐Ti) by the in situ growth of MnO_2_ on *g*‐C_3_N_4_ (Figure 10F,G).^[^ ^70^ ^]^ The photocatalytic property of MnO_2_/*g*‐C_3_N_4_‐Ti was evaluated by electrochemical impedance spectroscopy (EIS) measurements. The EIS results shown in Figure 10H revealed that MnO_2_/*g*‐C_3_N_4_‐Ti had the smallest arc among all samples, indicating the lowest charge transfer resistance and greatest electron/hole separation efficiency. Moreover, MnO_2_/*g*‐C_3_N_4_‐Ti more easily triggers more carriers and promotes charge separation under light irradiation. According to the curves shown in Figure 10I, an increase of the photoconversion efficiency of MnO_2_/*g*‐C_3_N_4_ of 21.11% compared with *g*‐C_3_N_4_ was calculated, which was due to the acceleration of photoinduced charges and the blocked recombination of electron–hole pairs, facilitating ROS‐induced photodynamic antibacterial therapy under visible light irradiation. The MnO_2_ weight ratio was related to the photocatalyst activity of the Z‐scheme heterojunction MnO_2_/*g*‐C_3_N_4_. Too high or too low MnO_2_ loading is detrimental to the photocatalytic performance, as less MnO_2_ is related to less charge trapping sites, while excess results in the conversion of trapping sites to recombination sites. Thus, the resulting PDT enhancement was utilized for sterilization. Compared with *g*‐C_3_N_4_‐Ti, the photo‐triggered antibacterial efficacy against *S. aureus* and *E. coli* of MnO_2_/*g*‐C_3_N_4_‐Ti reached 99.96% and 99.26%, respectively (Figure 10K,L). Apart from that, severe damage of *E. coli* under illumination is displayed in the corresponding SEM images (Figure 10J), indicating enhanced PDT after the introduction of the heterojunction. In addition, the PDT efficiency was further enhanced through the regulation of the GSH level by MnO_2_/*g*‐C_3_N_4_‐Ti (Figure 10M,N).

**Figure 11:**
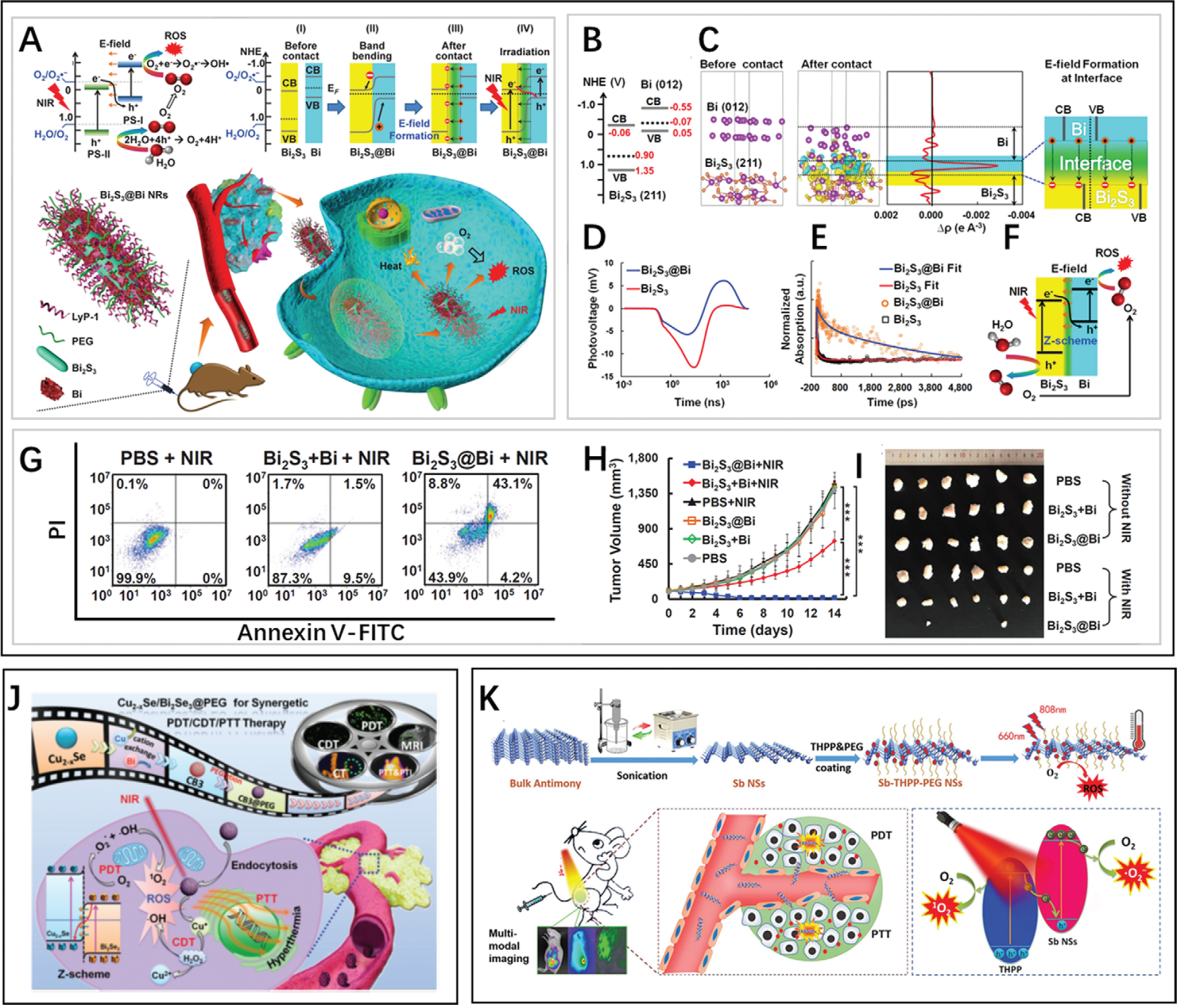
A) Principle of the phototherapeutic effect of Bi_2_S_3_@Bi nanoplatforms with Z‐scheme heterostructure. B) Band structures of Bi and Bi_2_S_3_ obtained by DFT calculation. C) CDD calculated by density functional theory before and after the combination, and planar‐averaged CDDs of the surfaces of Bi (012) and Bi_2_S_3_ (211) in Z direction; yellow areas indicate electron accumulation, and cyan areas indicate electron depletion. D) TPV spectra of Bi_2_S_3_ and Bi_2_S_3_@Bi. E) TAS kinetics of Bi_2_S_3_ and Bi_2_S_3_@Bi at 694 nm. F) Illustration of ROS generation in the Z‐scheme heterojunction Bi_2_S_3_@Bi. G) Flow cytometric assay of 4T1 cells with Annexin V–FITC/PI. H,I) Tumor growth curves and tumor photographs of diverse treatment groups. Reproduced with permission.^[^ ^71^ ^]^ Copyright 2020, Wiley‐VCH. J) Schematic illustration of the preparation and multimodal imaging‐guided cancer theranostics of Cu_2‐x_Se/Bi_2_Se_3_@PEG. Reproduced with permission.^[^ ^72^ ^]^ Copyright 2020, Royal Society of Chemistry. K) Schematic illustration of the preparation and multimodal imaging‐guided cancer theranostics of Sb‐THPP‐PEG NSs. Reproduced with permission.^[^ ^13b^ ^]^ Copyright 2020, Wiley‐VCH.

Furthermore, Cheng and co‐workers synthesized Z‐scheme hetero‐structured photocatalysts and PT agents based on Bi_2_S_3_@Bi nanorods (NRs) via combining the solvothermal method with hydrazine treatment.^[^ ^71^ ^]^ In particular, due to the different Fermi levels of Bi_2_S_3_ and Bi, charge migration after the contact of Bi_2_S_3_ and Bi results in the formation of a built‐in electric field (E‐field). This facilitates the diffusion of electrons from the CB of Bi_2_S_3_ to holes in the VB of Bi, resulting in higher redox potentials for the formation of ROS (**Figure** **11**A,B). The calculated charge density difference (CDD) are shown in Figure 11C, revealing the electron transfer and the presence of the E‐field. Through transient photovoltage (TPV; Figure 11D) and transient absorption spectroscopy (TAS; Figure 11E), the process of charge migration was further illustrated, which suggested the successful formation of the Bi_2_S_3_@Bi Z‐scheme heterojunction architecture. Thus, under NIR irradiation, O_2_ and ROS were generated in the VB of Bi_2_S_3_ and in the CB of Bi, respectively (Figure 11F), by spatiotemporal synchronization to achieve phototherapy of tumors under hypoxic conditions. In an in vitro assay, the Bi_2_S_3_@Bi NR heterojunction presented excellent biocompatibility under dark conditions. Flow cytometry analysis (Figure 11G) revealed that 56.1% of apoptotic/necrotic cells were induced by the Bi_2_S_3_@Bi NRs exposed to an 808 nm laser, suggesting NIR‐triggered cytotoxicity. Moreover, superior tumor inhibition in vivo demonstrated their general potential for clinical application (Figure 11H,I). In 2021, Wang et al. reported Cu_2−x_Se/Bi_2_Se_3_ with Z‐scheme heterojunction for anticancer treatment,^[^ ^72^ ^]^ and the band structure after optimization displayed 6‐times greater ROS generation than the pure sample (Figure 11J). Likewise, Kang and co‐workers designed an antimonene nanosheet‐based Z‐scheme heterostructure,^[^ ^13b^ ^]^ which also facilitated improved photonic therapy due to the Z‐scheme heterojunction (Figure 11K).

Compared with type II heterojunction, some of the excited electrons and holes in the Z‐scheme heterojunction recombine at the interface, resulting in a certain degree of waste. However, the separated electrons and holes were located at higher levels of CB and VB of Z‐scheme heterojunction, in which the potential energy of electron‐hole catalytic REDOX reaction is increased. Hence, the type of catalytic reactions and therapeutic products are greatly expanded.

### 4.3 P‐N Heterojunction

Similar to traditional type II heterojunction, the band structures (VB and CB) of the two catalysts are interlaced and the transfer path of excited electrons and holes were as same as that of type II heterojunction. However, due to the presence of electric field in the interface between p‐type semiconductor and N‐type semiconductor, the transfer directionality and efficiency of excited electrons and holes at p‐n heterojunction are much higher than that of conventional type II heterojunction. Meanwhile, the defect of p‐n node is the same as that of type II heterojunction. Although the separation efficiency of excited electrons and holes is improved, the REDOX potential is reduced, which reduces the ability of catalytic REDOX reaction.

**Figure 12:**
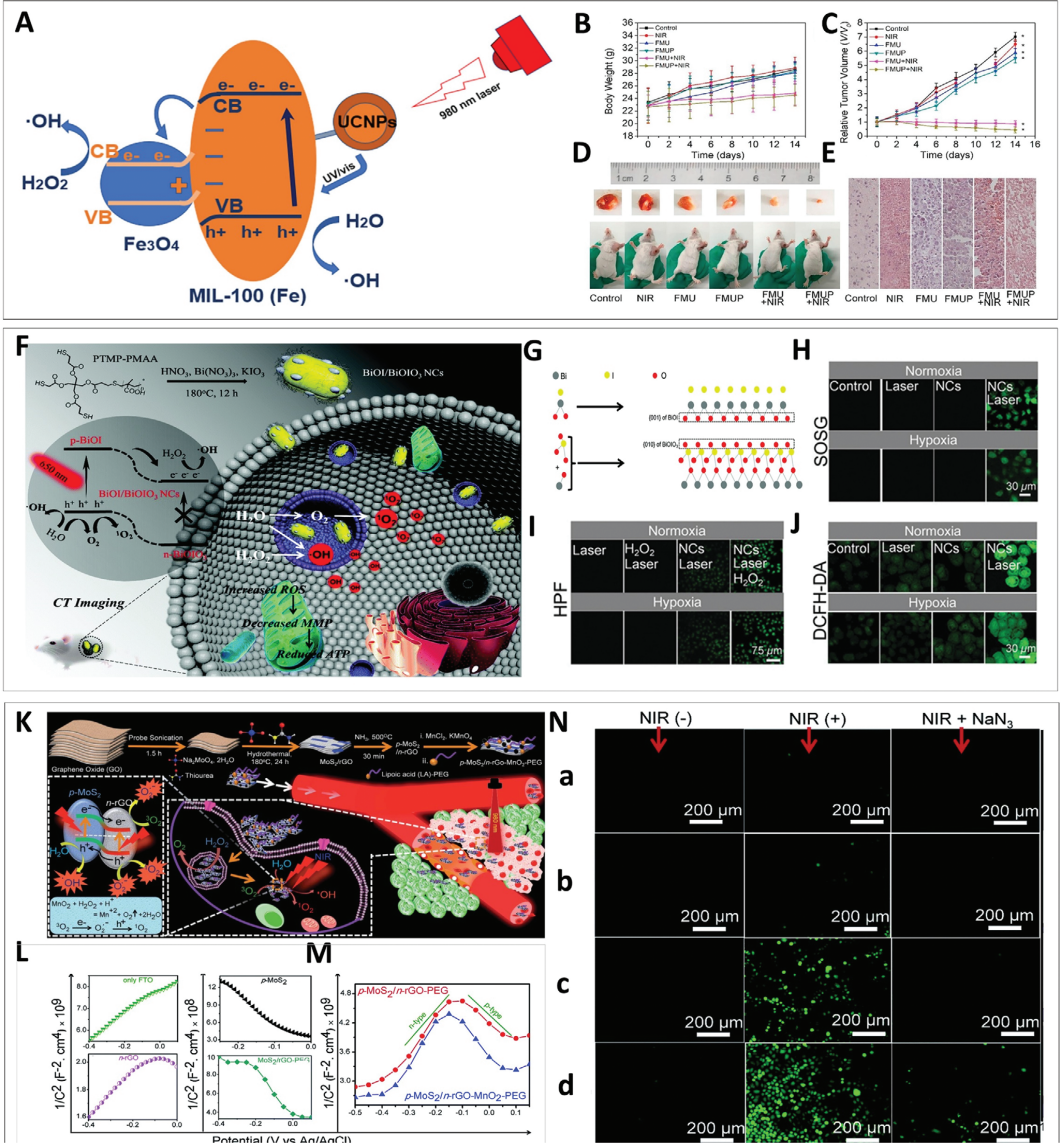
A) Illustration of photocatalysis mediated by Fe_3_O_4_@MIL‐100(Fe). B) Bodyweight curves and C) tumor growth curves of mice in different treatment groups. D) Photographs of tumors dissected from mice treated under diverse conditions, and photographs of the corresponding mice. E) Tumor tissue stained with H&E after the treatment. Reproduced with permission.^[^ ^73^ ^]^ Copyright 2018, Elsevier. F) Schematic diagram of the preparation and therapeutic mechanism of BiOI/BiOIO_3_. G) Formation mechanism of BiOI/BiOIO_3_. CLSM images of HeLa cells after staining with H) SOSG, I) HPF, and J) DCFH‐DA under different treatments. Reproduced with permission.^[^ ^74^ ^]^ Copyright 2019, Royal Society of Chemistry. K) Diagrammatic drawing of the synthesis and function of p‐MoS_2_/n‐rGO—MnO_2_‐PEG. L) Mott–Schottky plots of the bare FTO glass substrate, p‐type MoS_2_, n‐rGO, and MoS_2_/rGO‐PEG. M) Overlay of the Mott–Schottky plots of p‐MoS_2_/n‐rGO‐PEG and p‐MoS_2_/n‐rGO—MnO_2_‐PEG. N) Fluorescence microscopy images of HeLa cells incubated with a) DCFH‐DA, b) DCFH‐DA + MoS_2_/rGO‐PEG, c) DCFH‐DA + p‐MoS_2_/n‐rGO‐PEG, or d) DCFH‐DA + p‐MoS_2_/n‐rGO—MnO_2_‐PEG under NIR irradiation for 5 min and in the presence or absence of NaN_3_. Reproduced with permission.^[^ ^75^ ^]^ Copyright 2018, Royal Society of Chemistry.

To date, a lot of efforts have been placed on p‐n heterojunctions for PDT. For example, the integration of PDT and photochemotherapy was proposed by Wang and co‐workers through the construction of the Fe_3_O_4_@MIL‐100(Fe)‐UCNPs‐PEG (FMUP) nanoplatform.^[^ ^73^ ^]^ The Fe_3_O_4_@MIL‐100(Fe) (FM) heterojunction was obtained by a layer‐by‐layer assembly strategy, and the upconversion nanoparticles (UCNPs) were modified by covalent attachment, which enabled to make use of the advantages of NIR and UV/vis light (**Figure** **12**A). Based on the Hall test, the FM was proved to be both an n‐ and p‐type semiconductor, which implied the formation of an FM p‐n heterojunction. Accordingly, cytotoxic ·OH was produced by photoinduced charge transfer in the presence of the internal electric field and electron–hole pair separation by the p‐n heterojunction. In addition, UV/vis light, converted from NIR by UCNPs, could induce the Fe‐ion‐based Fenton reaction even in tumor microenvironment where the pH did not meet the requirements (pH = 3–4) to assist PDT in the production of apoptosis‐inducing ROS. In an in vivo assay, the gradual increase of the body weight over time in all groups showed good biosafety and no adverse drug reaction (Figure 12B). The obvious decrease in the tumor volume and the clear damage of cancer tissue suggested an excellent tumor‐killing ability of FMUP under light irradiation (Figure 12C–E).

In cancer treatment, hypoxia in the tumor site is another reason restricting PDT application. To overcome this limitation, Zhen et al. developed a p–n heterostructure to simultaneously achieve effective type‐I and O_2_ self‐supplying type II PDT by hydrothermal coupling of BiOI and BiOIO_3_.^[^ ^74^ ^]^ When BiOI/BiOIO_3_ was exposed to a 650 nm laser, the photoinduced electrons and holes accumulated in the CB of BiOIO_3_ and the VB of BiOI, respectively, according to their electronic band structure (Figure 12F), which was further improved due to the internal electric field. In the BiOI/BiOIO_3_ formation mechanism (Figure 12G), O_2_ was shared by BiOI and BiOIO_3_, resulting in an intimate contact, which significantly enhanced the migration of photoinduced charges. Irrespective of the hypoxic microenvironment of cancer, the clustered electrons converted H_2_O_2_ into most cytotoxic ·O, while the collective holes reacted with H_2_O to generate not only ·OH but also O_2_. O_2_ was further converted into toxic ^1^O_2_ to simultaneously realize the two types of PDT. Fluorescence analysis of cells verified this hypothesis because various cell probes treated with nanocomposites and laser (regardless of normoxia) exhibited bright fluorescence, which is indicative of ROS (Figure 12H–J). It should be noted that the fluorescence of hydroxyphenyl fluorescein (HPF, ·OH probe) in normoxia or hypoxia was stronger after addition of extra hydrogen peroxide, which proved ·OH generation in the absence of O_2_.

Regarding O_2_ self‐providing PDT via the Fenton reaction, Kapri et al. designed a p‐MoS_2_/n‐rGO–MnO_2_‐PEG nanocomposite as a p‐n heterojunction,^[^ ^75^ ^]^ which was fabricated through a series of methods, including liquid exfoliation and hydrothermal reaction (Figure 12K). The Mott–Schottky plots shown in Figure 12L,M revealed the p‐type nature of MoS_2_ and the n‐type nature of rGO. As for p‐MoS_2_/n‐rGO–MnO_2_‐PEG nanosheets, Mott–Schottky plots with an inverted “V‐shape” verified p‐n heterojunction characteristics. Acting as a photosensitizer, the p‐MoS_2_/n‐rGO–MnO_2_‐PEG was triggered under NIR irradiation so that the resulting separated charge carriers converted O_2_ into ROS. This hypothesis was proved by ROS detection in vitro. Specifically, DCFH‐DA was employed as an indicator to detect the ROS level in cells after different treatments. Figure 12N shows strong fluorescent intensity in the cells after treatment with p‐MoS_2_/n‐rGO–MnO_2_‐PEG plus irradiation, while an obvious reduction in fluorescence was detected after the addition of NaN_3_ scavenger, suggesting outstanding ROS generation by the p‐n heterojunction. In addition, the further fluorescence enhancement in the group of p‐MoS_2_/n‐rGO–MnO_2_‐PEG plus irradiation proved that self‐providing of O_2_ improved PDT.

### 4.4 S–M Heterojunction

In contrast to semiconductor‐semiconductor heterojunctions, there is a large class of heterojunctions that are composed of semiconductors and metals. Semiconductor–metal heterojunctions mainly include Schottky, Ohmic, and LSPR–mediated junctions. Schottky junctions are built by connecting a metal with higher work function (*Φ*) and an n‐type semiconductor with lower *Φ* or by connecting a metal with lower *Φ* and a p‐type semiconductor with higher *Φ*, Which could facilitate the separation and trap the excited electrons and prevent the recombination of electron‐hole pairs. Contrary to Schottky contacts, Ohmic junctions are formed by connecting an n‐type semiconductor with lower *Φ* and a metal with higher *Φ* or by connecting a p‐type semiconductor with higher *Φ* and a metal with lower *Φ*, which are bad for the separation of electron‐hole pairs. In addition, the LSPR effect promotes the generation of photoinduced electrons with high energy (hot electrons) in the noble metal. Then, the hot electrons cross the Schottky barrier and are injected into the semiconductor, while the photoinduced holes remain in the metal, which provides more negative electrons. Although semiconductor–metal heterojunctions have great potential in the field of catalysis, the strict requirements for semiconductors and metals have severely limited its development in the biomedical field.

**Figure 13:**
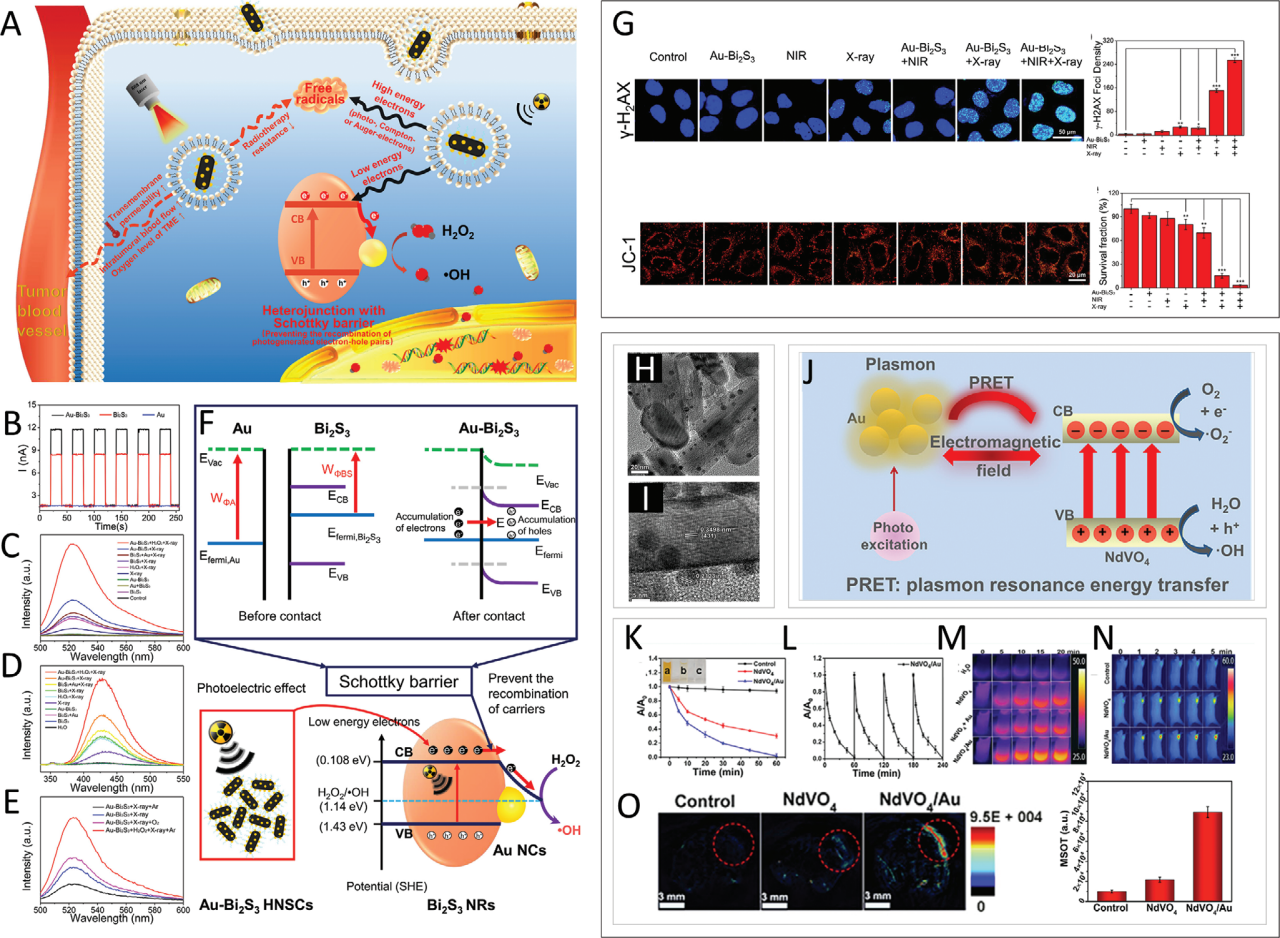
A) Principle of the Au—Bi_2_S_3_‐mediated therapeutic process. B) Photocurrent responses of different samples to the switch on/off of X‐ray irradiation. C) Free radical measurements under diverse conditions. D) Hydroxyl radical measurements of different samples. E) Free radical measurement of Au‐Bi_2_S_3_ under X‐ray irradiation and different oxygen environments. F) Proposed mechanism of Schottky‐type heterostructures Au—Bi_2_S_3_ for the increased free radical production under X‐ray irradiation. G) Fluorescence images of Hela cells stained with Hoechst 33 342 (blue) and *γ*‐H_2_AX (green) and labelled with JC‐1. Reproduced with permission.^[^ ^76^ ^]^ Copyright 2019, American Chemical Society. H) TEM and I) HRTEM images of NdVO_4_/Au. J) Illustration of the photocatalytic performance of NdVO_4_/Au. K) Photo‐degradation of methyl orange under different conditions. L) Cycling test of the photoinduced decomposition of methyl orange in the presence of NdVO_4_/Au. M) Thermal images of samples irradiated by an 808 nm laser for different times. N) Thermal images of tumor‐bearing mice under different treatments and 808‐nm laser irradiation for different times. O) PA images and values of the tumor site at 12 h post‐injection with saline, NdVO, and NdVO_4_/Au. Reproduced with permission.^[^ ^77^ ^]^ Copyright 2019, Elsevier.

Recently, Wang et al. reported Au—Bi_2_S_3_ heterojunction nanoparticles^[^ ^76^ ^]^ prepared by the in situ growth of Au on the surface of Bi_2_S_3_ (**Figure** **13**A). Due to the construction of the Schottky barrier between Bi_2_S_3_ and Au, the electrons transferred to the CB under the irradiation of X‐ray could be trapped in Schottky barrier, which inhibited the combination of electron‐hole pairs and facilitated H_2_O_2_ as electron acceptor to be decomposed into ·OH for tumor destruction. The catalytic performance was analyzed by measuring the photocurrent under X‐ray irradiation, showing that the current density induced by Au—Bi_2_S_3_ was 1.5 times as high as that obtained by pristine Bi_2_S_3_, which directly proved that the Au—Bi_2_S_3_ heterojunction enhanced the separation of X‐ray‐excited electrons and holes (Figure 13B). The photocatalytic ability of the Au—Bi_2_S_3_ heterojunction in the generation of free radicals is 1.6 times as high as that of the Au and Bi_2_S_3_ mixture under the same conditions (Figure 13C), demonstrating that the formation of the heterojunction strengthened the photocatalytic effect. Furthermore, the triggered low energy electrons in the Au—Bi_2_S_3_ heterojunction are captured by Au via the electron trap of the Schottky barrier to react with overexpressed H_2_O_2_ and generate ·OH, which was verified by the significant fluorescence increase of terephthalate (specific indicator for ·OH) after hydroxyl radical detection (Figure 13D). Interestingly, Figure 13E reveals a significant increase of free radicals in the group of Au—Bi_2_S_3_+H_2_O_2_+X‐ray+Ar, manifesting that the photoconversion of H_2_O_2_ to ROS broke the limitation by hypoxia. To evaluate the anticancer activity, DNA damage was analyzed by *γ*‐H_2_AX foci detection. Figure 13G shows that the green fluorescence of cells co‐incubated with Au—Bi_2_S_3_ composite plus X‐ray irradiation was 5.6 times stronger than in the group only exposed to X‐ray irradiation, demonstrating that the radiosensitization enhancement was caused by the heterojunction. Interestingly, the green fluorescence was further strengthened when the cells were exposed to X‐ray and NIR irradiation after incubation with Au—Bi_2_S_3_. This was attributed to the improved uptake of Au—Bi_2_S_3_ by the cells due to the change in transmembrane permeability under the PT effect of Au—Bi_2_S_3_ nanoparticles exposed to NIR irradiation. The JC‐1 assay proved cell damage resulting from free radicals by the loss of the mitochondrial membrane potential. Overall, phototherapy based on Au—Bi_2_S_3_ is independent of oxygen so that it is more suitable for treating cancer in hypoxic environment than conventional PDT.

LSPR is a useful method that plays a significant role in improving photocatalysis. Chang et al. combined the hydrothermal method with a reduction to prepare NdVO_4_/Au heterojunction nanocrystals as plasmonic photocatalysts,^[^ ^77^ ^]^ and the corresponding TEM and HR–TEM images are presented in Figure 13H,I. Due to the LSPR effect, the plasmonic metal Au interacted strongly with the laser, which resulted in an enhanced light absorption and the formation of an electromagnetic field. Therefore, under laser irradiation, the LSPR‐triggered plasmon resonance energy transfer (PRET) from Au to NdVO_4_ surmounted the wide bandgap of NdVO_4_ (Figure 13J), which allowed the formation of a considerable number of electron–hole pairs to participate in redox reactions so that the photocatalytic performance was strengthened for ROS generation. Accordingly, the solar‐driven photocatalytic activity was tested by analyzing the degradation of methyl orange. Figure 13K,L shows that methyl orange was almost completely decomposed in the presence of NdVO_4_/Au under solar irradiation, and no obvious deactivation of the photodegradation ability was detected even after four consecutive cycles, which further demonstrated the outstanding photocatalytic performance of NdVO_4_/Au. In addition, the enhanced PT conversion efficiency and improved thermal expansion effect endowed the nanocomposites with the potential of PT/PA imaging. The real‐time PT image of mice injected with NdVO_4_ or NdVO_4_/Au under NIR laser irradiation (Figure 13F,G) showed an obvious variation of the temperature after NdVO_4_/Au treatment, indicating the superior potential of NdVO_4_/Au as a PT agent. Among different treatments, NdVO_4_/Au treatment of U14‐tumor‐bearing mice exhibited the strongest photoacoustic (PA) signal, indicating the good PA imaging ability of NdVO_4_/Au (Figure 13H,I).

**Figure 14:**
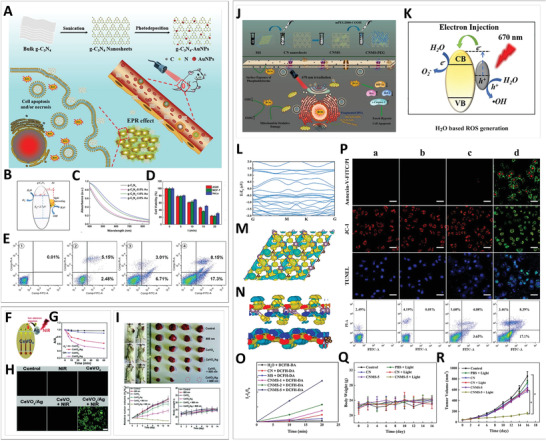
A) Illustration of *g*‐C_3_N_4_−Au‐based PDT. B) High‐angle annular dark field‐scanning transmission electron microscope (HAADF‐STEM) image of *g*‐C_3_N_4_‐Au (scale bar = 200 nm). C) UV–vis spectrum of g‐C_3_N_4_‐Au. D) Anticancer effect of *g*‐C_3_N_4_‐1.0% Au in a hypoxic atmosphere. E) Flow cytometry images of MCF‐7 after different treatments in a hypoxic atmosphere: ① MCF‐7; ② MCF‐7 + light; ③ MCF‐7 + *g*‐C_3_N_4_‐1.0% Au; ④ MCF‐7 + *g*‐C_3_N_4_‐1.0% Au + 20 min light irradiation (*g*‐C_3_N_4_‐1.0% Au concentration: 200 µg mL^−1^). Reproduced with permission.^[^ ^78^ ^]^ Copyright 2021, American Chemical Society. F) Schematic illustration of enhanced phototherapy based on CeVO_4_/Ag. G) ROS generation capability of CeVO_4_ and CeVO_4_/Ag. H) Fluorescence images of HeLa cells stained with DCFH‐DA under different treatments. I) Representative images showing the changes of tumor volume and body weight in an in vitro assay. Reproduced with permission.^[^ ^79^ ^]^ Copyright 2019, Royal Society of Chemistry. J) Schematic diagram of CN@MS heterojunction‐mediated PDT. K) ROS generation based on the CN@MS heterojunction. L) Band structure of the CN@MS heterojunction. CDD M) top and N) side view of the CN@MS heterojunction. The upper layer is MS and the lower layer is CN. Purple, yellow, brown, and silver indicate Mo, S, C, and N atoms, respectively. The isosurfaces were set to 1.23 × 10^−6^ e Å^−3^. O) Fluorescence intensity of DCFH after different treatments. P) Fluorescence images and the corresponding flow cytometry analysis of (a) cell; (b) cell + light; (c) cell + CN@MS; and (d) cell + CN@MS + light after different treatments (scale bar = 50 µm). Changes of the Q) body weight and R) tumor growth curves under different conditions. Reproduced with permission.^[^ ^80^ ^]^ Copyright 2019, Elsevier.

Moreover, Dai et al. fabricated *g*‐C_3_N_4_ coated with Au nanoparticles by the joint approach of liquid‐phase exfoliation and photo‐deposition (**Figure** **14**A).^[^ ^78^ ^]^ As a semiconductor material with great photocatalytic activity, *g*‐C_3_N_4_ has a wide bandgap of 459 nm, which severely hinders its application in PDT. They reported that Au makes use of the laser energy to move and accumulate electrons in the CB of *g*‐C_3_N_4_, which improves the separation efficiency of photoexcited charges and facilitates ROS generation from H_2_O (Figure 14B). In addition, on account of the Au modification, the photo‐absorption range of the *g*‐C_3_N_4_/Au heterojunction could be extended to the NIR region (Figure 14C). When the Au content reached 1.0%, *g*‐C_3_N_4_/Au exhibited the best photocatalytic ability to induce tumor cell death even in the absence of oxygen (Figure 14D). This was ascribed to the high number of ROS formed from H_2_O by the *g*‐C_3_N_4_/Au heterojunction, which was confirmed by the flow cytometry assay (Figure 14E).

Compared with Au, Ag exhibits many advantages, including the relatively low cost and extraordinary plasmonic properties. Chang et al. reported a CeVO_4_/Ag nanohybrid heterojunction with outstanding photoconversion capability for ROS production.^[^ ^79^ ^]^ Under NIR laser irradiation, hot electrons formed after absorption of resonant photons by Ag and moved into the CB of CeVO_4_ to effectively separate electrons and holes and facilitate the generation of O_2_^−^ and ·OH in the CB and VB of CeVO_4_, respectively (Figure 14F). As shown in Figure 14G, the gradual decrease in the absorption intensity of 1,3‐diphenylisobenzofuran (DPBF) and methylene blue over time indicated the continuous ROS generation after laser irradiation of the CeVO_4_/Ag heterojunction. According to Figure 14H, enhanced ROS generation was proved by the changes in the intracellular fluorescence of 2′,7′‐dichlorodihydrofluorescein diacetate (DCFH‐DA), and green fluorescence indicated the superb ROS production ability of CeVO_4_/Ag via photocatalysis. The ROS‐mediated antitumor activity was further analyzed by in vivo experiments, as displayed in Figure 14I, showing that the tumor suppression ratio after CeVO_4_/Ag plus NIR illumination treatment was superior to that of other treatment groups, implying the enormous potential of the CeVO_4_/Ag heterojunction in anti‐tumor treatment.

Apart from noble metals, metallic transition metal dichalcogenides can be employed to transfer photoinduced electrons. Dai and co‐workers integrated graphitic carbon nitride (CN) and metallic molybdenum disulfide (MS) into a CN@MS heterojunction system^[^ ^80^ ^]^ in which MS acted as the antenna to respond to the incident light and induce the injection of photogenerated electrons into the CN for electron–hole separation (Figure 14J,K). To prove the successful construction of this heterostructure, DFT calculations were performed. Figure 14L shows that the calculated valence band maximum (VBM) and the conduction band minimum (CBM) were located at K and G points, respectively, indicating that pure CN has the characteristics of an indirect band‐gap semiconductor. The CDD plot demonstrated that the heterojunction formation was contributed by the electron rearrangement at the stacking interface and the noncontact surface (Figure 14M,N). Figure 14O shows that massive ROS generation resulted from the construction of the CN@MS heterostructure due to fluorescence quenching of DCFH‐DA. An in vitro assay confirmed ROS‐induced cell apoptosis via fluorescent staining and flow cytometry (Figure 14P). To further assess the anticancer effect, the experiment was conducted in vivo. Figure 14Q,R shows body weight and tumor growth curves, which exhibited little fluctuation and thus good biosafety and a good therapeutic effect, indicating that the CN@MS heterojunction is a promising candidate for clinical application.

## 5 Summary and Future Perspectives

As an effective strategy to enhance the catalytic efficacy, heterojunctions have been intensely studied for application in the energy conversion field, yet comparable studies in biomedicine are just in their infancy but promise huge potential. Over decades, some heterojunction‐based catalytic systems have been designed for biomedical applications (**Table** **1**). Herein, recent achievements and the development of heterojunctions in the medical field are introduced through some examples, including the design procedures, synthetic methods, specific applications, and treatment efficacy, showing that the heterojunction‐based catalytic system has promising biomedical properties and prospects. To achieve the desired effect, the requirements, including the rational alignment of energy bands and ideal migration of photogenerated electron–hole pairs, the shape uniformity preparing through simple, economical, but efficient ways, need to be met. And the clinical application of heterojunction medicine has never appeared, because several problems have not been solved yet. 1) Basically, the balance between biosafety and therapeutic efficacy of heterojunctions should be realized. The higher doses of heterojunctions, the higher potential of side effects. Further research needs to enhance the therapeutic effect of per unit dose, such as the increase in the number of active sites and the targeting to specific lesions. Development of biodegradable and metabolizable materials is another way. 2) Current studies almost focus on the common, existing catalytic reactions. We can explore other potential reactions of heterojunctions (whether reported or not in biomedical application), preparing for possible application or adverse reactions. Apart from the heterojunction effect of the entire composite, other properties, such as the bioimaging ability, determined by the composition of the materials should be focused on to realize multi‐functional integration. 3) Turning basic research into clinical application require a large amount of time and money. To speed up the process, we believe that the in‐depth theoretical research can guide the future research directions of heterojunction nanomedicine.

**Table 1:** List of different heterojunctions for ROS catalysis in biomedicine field

Heterojunction structure	Materials	Catalytic behaviors
Type‐II heterojunctions	BiOI/Bi_2_S_3_^[^ ^64^ ^]^	O_2_ → **·**O_2_^−^ **;** OH^−^ → **·**OH
Type‐II heterojunctions	WO_2.9_/WSe_2_^[^ ^65^ ^]^	H_2_O_2_ → **·**OH
Type‐II heterojunctions	As‐As_x_O_y_^[^ ^68^ ^]^	O_2_ → **·**O_2_^−^ **;** O_2_ → ^1^O_2_ **;** GSH → GSSG
Z‐scheme heterojunctions	FeS/Fe_2_O_3_^[^ ^58^ ^]^	H_2_O_2_ → **·**OH**;** OH^−^→ **·**OH**;** O_2_ → **·**O_2_^−^ **;** GSH → GSSG
Z‐scheme heterojunctions	FeTiO_3_/Fe_2_O_3_^[^ ^69^ ^]^	H_2_O_2_ → **·**OH**;** OH^−^→ **·**OH**;** O_2_ → **·**O_2_^−^
Z‐scheme heterojunctions	MnO_2_/*g*‐C_3_N_4_^[^ ^70^ ^]^	OH^−^→ **·**OH**;** O_2_ → **·**O_2_^−^ **;** O_2_ → ^1^O_2_ **;**
Z‐scheme heterojunctions	Bi_2_S_3_/Bi^[^ ^71^ ^]^	H_2_O → O_2_ **;** O_2_ → **·**O_2_^−^
Z‐scheme heterojunctions	Cu_2−x_Se/Bi_2_Se_3_^[^ ^72^ ^]^	O_2_ → ^1^O_2_
Z‐scheme heterojunctions	Sb/THPP(5,10,15,20‐Tetrakis(4‐hydroxy‐phenyl)‐21H,12Hporphine)^[^ ^13b^ ^]^	O_2_ → **·**O_2_^−^ **;** O_2_ → ^1^O_2_
P‐N heterojunction	Fe_3_O_4_@MIL‐100(Fe)^[^ ^73^ ^]^	H_2_O_2_ → **·**OH**;** H_2_O → **·**OH
P‐N heterojunction	BiOI/BiOIO_3_^[^ ^74^ ^]^	H_2_O_2_ → **·**OH**;** H_2_O → **·**OH**;** H_2_O → O_2_ **;** O_2_ → ^1^O_2_ **;**
P‐N heterojunction	MoS_2_/n‐rGO—MnO_2_^[^ ^75^ ^]^	O_2_ → **·**O_2_^−^ **; ·**O_2_^−^ →^1^O_2_ **;** H_2_O → **·**OH
S‐M heterojunction	Au/Bi_2_S_3_^[^ ^76^ ^]^	H_2_O_2_ → **·**OH
S‐M heterojunction	NdVO_4_/Au^[^ ^77^ ^]^	O_2_ → **·**O_2_^−^ **;** H_2_O → **·**OH
S‐M heterojunction	*g*‐C_3_N_4_/Au^[^ ^78^ ^]^	H_2_O → **·**O_2_^−^ **;** H_2_O → **·**OH
S‐M heterojunction	CeVO_4_/Ag^[^ ^79^ ^]^	O_2_ → **·**O_2_^−^ **;** H_2_O → **·**OH
S‐M heterojunction	graphitic carbon nitride/ metallic molybdenum disulfide^[^ ^80^ ^]^	H_2_O → **·**O_2_^−^ **;** H_2_O → **·**OH

### 5.1 Enhance Catalytic Efficiency and Biosafety

The toxicity of heterojunctions to normal tissue restricts the in vivo application. Compared with the uniform distribution of catalytic sites of homogeneous photocatalysts, active sites of heterogeneous photocatalysts restrict the photocatalytic capability. Thereby, enlarging the specific surface area is one of the effective strategies to achieve the exposure of sufficient active sites. Compared with conventional 3D materials, the surface effects and the huge specific surface area of the emerging 2D structures, due to the atomic layer thickness, not only present the unique photoelectric performances of materials but also provide a considerable number of active sites for an increased responsiveness.^[^ ^81^ ^]^ And the bandgap of 2D materials can be controlled by changing the number of layers to achieve a response to different wavelengths for various applications.^[^ ^81^, ^82^ ^]^ Many examples proved that some heterojunctions can uptake the advantages and exhibit better function than the only use of the component. BP, a typical 2D material used for tumor treatment, has an adjustable bandgap. When the thickness decreases from five layers to a single layer, the bandgap increases from 0.59 to 1.51 eV,^[^ ^83^ ^]^ enabling BP to absorb light in the UV–vis–NIR region and overcome the shortcomings of Si‐based photodetectors in response to NIR laser irradiation. And it is reported that photodetectors with high responsivity by the formation of a heterojunction of BP and Si.^[^ ^84^ ^]^ The key role 2D heterojunction materials playing in the technology of photoelectrical detection makes us speculate that band‐gap‐matched heterojunction materials based on 2D structures, including but not limited to BP, may exhibit better properties for biomedical use than conventional materials in the medical field.

In addition to the controlled bandgap, BP is profitable for practical application due to the biodegradability of it and the biosafety of the degradation product, phosphate.^[^ ^85^ ^]^ Also, quantum dots with the size of several nanometers are safer than other materials with large size, because they can be eliminated through kidney.^[^ ^86^ ^]^ Similar to other agents, targeting promises better therapeutic results with less dose. In a word, heterojunctions consisting of biodegradable materials and targeting systems have a larger potential to turn into clinic.

### 5.2 Expand Catalytic Reaction and Application Types

With the continuous research and development of nanomedicine, multimodal therapies have been utilized for antitumor therapies. Apart from ROS generation, heterojunctions can be utilized in catalyzing several biological and chemical reactions due to the controlled band structure, and therefore adopted for many therapeutic methods due to their superior optical properties.

Gas therapy, as an emerging antitumor treatment, is used to kill cancer cells through the specific accumulation of toxic gas at the tumor site. However, this treatment still faces challenges because of the uncontrollable and ineffective release of therapeutic gas in vivo. To solve this problem, Wang and co‐workers proposed a CO nanogenerator synthesized by partially oxidized SnS_2_, which had an excellent antitumor effect.^[^ ^87^ ^]^ Also, hydrogen treatment possesses a superb regulation function in physiology and pathology.^[^ ^88^ ^]^ It can be employed to not only inhibit tumors,^[^ ^89^ ^]^ but also reduce ischemia reperfusion injury,^[^ ^90^ ^]^ and improve rheumatoid arthritis.^[^ ^91^ ^]^ To hinder the adverse nature of hydrogen (e.g., uncontrollable diffusion),^[^ ^92^ ^]^ the heterojunction photocatalyst for hydrogen generation through the coupling of graphene and g‐C3N4 has been fabricated,^[^ ^93^ ^]^ taking advantage of the optical properties of graphene for the effective separation of photoinduced charges.^[^ ^94^ ^]^ The results show that as a strategy to improve the permeability and photocatalytic efficiency of gases, the heterojunction nanoplatform is a promising candidate to be introduced in the field of gas therapy to control the gas production with high accuracy.

In addition, heterojunctions can also play a role in PT therapy. Deng et al. introduced the CuS@Cu_2_S@Au nanohybrid as a PT agent.^[^ ^95^ ^]^ After the formation of Cu_2_S on the surface of CuS and the modification of Au, the energy band changed and more circuit paths are provided for charge migration, which contributed to increasing the PT conversion efficiency. Moreover, Cui et al. reported a composite resulting from coupling Au and Cu_7_S_4_ to ablate tumors via PTT.^[^ ^96^ ^]^ After the formation of Cu_7_S_4_‐Au heterodimers, the LSPR absorption was changed, and the response of this system was optimized to 808 nm laser irradiation so that the PT effect was strengthened.

### 5.3 Equip with Theoretical Strategies

Theoretical strategies such as first‐principles calculations and molecular dynamics simulations have been widely adopted in material design,^[^ ^97^ ^]^ functional evaluation,^[^ ^98^ ^]^ and mechanism analysis.^[^ ^99^ ^]^ Combination of experimental research and theoretical calculations can improve the efficiency of heterojunction development and save costs. In addition, the application of theoretical calculations can provide an in‐depth supplement to the mechanism study of heterojunction photocatalysts. At present, many constructive results have been achieved in theoretical calculations based on the vertical heterojunction stacked by van der Waals forces.^[^ ^100^ ^]^ However, due to the more complicated factors at the interface in the transverse heterojunction, the existing theoretical researches are limited to graphene‐like materials and 2D transition metal sulfides.^[^ ^101^ ^]^ Therefore, to better provide theoretical support for the development of heterojunction, extending the scope of application through reasonable construction of the initial structure and optimization of the parameters has become a necessary matter.

In summary, the recent progress of heterostructure nanomedicine is presented in this review. This field of medicine provides an effective approach to optimize anti‐tumor effects. We hope that this review provides a new perspective for the application of heterostructure nanomedicine to advance the further development of this novel field of medicine in biomedicine.

## Conflict of Interest

The authors declare no conflict of interest.
